# Incidence and Tracking of *Escherichia coli* O157:H7 in a Major Produce Production Region in California

**DOI:** 10.1371/journal.pone.0001159

**Published:** 2007-11-14

**Authors:** Michael Cooley, Diana Carychao, Leta Crawford-Miksza, Michele T. Jay, Carol Myers, Christopher Rose, Christine Keys, Jeff Farrar, Robert E. Mandrell

**Affiliations:** 1 Produce Safety and Microbiology Research Unit, United States Department of Agriculture-Agricultural Research Service, Western Regional Research Center, Albany, California, United States of America; 2 California Department of Health Services, Food and Drug Laboratory Branch, Richmond, California, United States of America; 3 California Department of Health Services, Food and Drug Branch, California, United States of America; 4 Central Coast Regional Water Quality Control Board, California Environmental Protection Agency, San Louis Obispo, California, United States of America; 5 Food and Drug Administration/Center for Food Safety and Applied Nutrition (CFSAN)/Office of Plant and Dairy Foods (OPDF)/DMS, College Park, Maryland, United States of America; The Research Institute for Children, United States of America

## Abstract

Fresh vegetables have become associated with outbreaks caused by *Escherichia coli* O157:H7 (EcO157). Between 1995–2006, 22 produce outbreaks were documented in the United States, with nearly half traced to lettuce or spinach grown in California. Outbreaks between 2002 and 2006 induced investigations of possible sources of pre-harvest contamination on implicated farms in the Salinas and San Juan valleys of California, and a survey of the Salinas watershed. EcO157 was isolated at least once from 15 of 22 different watershed sites over a 19 month period. The incidence of EcO157 increased significantly when heavy rain caused an increased flow rate in the rivers. Approximately 1000 EcO157 isolates obtained from cultures of>100 individual samples were typed using Multi-Locus Variable-number-tandem-repeat Analysis (MLVA) to assist in identifying potential fate and transport of EcO157 in this region. A subset of these environmental isolates were typed by Pulse Field Gel Electrophoresis (PFGE) in order to make comparisons with human clinical isolates associated with outbreak and sporadic illness. Recurrence of identical and closely related EcO157 strains from specific locations in the Salinas and San Juan valleys suggests that transport of the pathogen is usually restricted. In a preliminary study, EcO157 was detected in water at multiple locations in a low-flow creek only within 135 meters of a point source. However, possible transport up to 32 km was detected during periods of higher water flow associated with flooding. During the 2006 baby spinach outbreak investigation, transport was also detected where water was unlikely to be involved. These results indicate that contamination of the environment is a dynamic process involving multiple sources and methods of transport. Intensive studies of the sources, incidence, fate and transport of EcO157 near produce production are required to determine the mechanisms of pre-harvest contamination and potential risks for human illness.

## Introduction

*Escherichia coli* O157:H7 (EcO157) is an enteric pathogen that can cause life threatening hemorrhagic colitis and, in very severe cases, hemolytic uremic syndrome [1]. It has been estimated that EcO157 causes approximately 73,000 illnesses in the United States each year. Between 1982 and 2002, 350 outbreaks caused by EcO157 were reported; 52% and 9% were caused by foodborne and waterborne sources, respectively [2]. Ground beef and produce were associated with 75 and 38 outbreaks, respectively, but the increased consumption of fresh fruits and vegetables in the U.S. appears to correlate with increased produce-associated outbreaks [2]. Since 1995, there have been 22 outbreaks of EcO157 associated with fresh lettuce or spinach, and 9 of these outbreaks have been traced to, or near, the Salinas Valley region of California [3]–[9]. The Salinas Valley region on the central coast of California is the major leafy vegetable producer in the US [10].

Contamination of produce can occur in the field by application of raw (or poorly composted) manure, exposure to contaminated water (irrigation or flooding) [11]–[14], or by deposition of feces by livestock or wild animals. Another possible, but unsubstantiated, source is contaminated dust from concentrated livestock or other animal operations. The presence of EcO157 in, or near, a raw produce production environment must be considered a potential risk factor for both sporadic- and outbreak-related human illness. Warm-blooded animals and invertebrates are known to carry EcO157 [15]–[24]. Persistence of EcO157 in the environment is variable and likely dependent upon a variety factors, including temperature, sunlight (ultraviolet), moisture, nutrients and possibly unknown factors that enhance or decrease amplification of EcO157. Recent studies indicate that EcO157 will survive in the field for four to eight weeks [13], [25]–[27], thus supporting the National Organic Program recommendation of a minimum of 120 days between manure application and harvest [28]. However, these and other studies also conclude that survival of EcO157 is improved at lower temperatures, in clay soil and in close association with roots [11], [25], [29], and in soil amended with cattle feces [30]. These results are consistent with the ability to isolate EcO157 from carrots 168 days after application of contaminated manure [12]. EcO157 present in agricultural environments are expected to be stressed, and possibly non-culturable, increasing the problem of measuring accurately the amount of pathogen in samples [31], [32]. However, in an unintended “natural” experiment of the virulence and survival of EcO157 for humans, the same EcO157 strain infecting a child exposed to the family garden fertilized with raw cow manure was isolated from the soil for approximately 70 days, and survived much better at ambient temperatures than at 4°C [27]. Nevertheless, contamination of ready-to-eat commodities with EcO157 is expected to be present at very low levels as evidenced by the rarity of outbreaks associated with raw produce compared to the volume of consumption.

The specific risk factors for in-field contamination, survival, and spread of EcO157 in the produce production environment remain unclear. Traceback investigations for one EcO157 outbreak reported by Washington state in 2002 and two outbreaks in California in 2003 identified a single farm in the Salinas Valley common to all three outbreaks [33]. The commodities (lettuce and spinach) involved, and the processing and distribution of these commodities varied between the outbreaks. The farm was the only factor common to all three outbreaks and was considered a likely source of the pre-harvest contaminated produce. Furthermore, an outbreak associated with a bagged mixed-produce product, including lettuce, occurred in Minnesota in September 2005 [34] and a large multi-state outbreak involving bagged spinach occurred in August/September, 2006 [6]. Traceback investigations for these outbreaks indicated that the produce was grown in the Salinas (Monterey County) and San Juan (San Benito County) valleys, respectively. A critical result in both the 2005 and 2006 bagged produce traceback investigations was the isolation of strains identical to the human outbreak strains from bagged produce obtained from patient's homes; a single lot number on the bags associated with the 2006 spinach outbreak was associated with a production run with product obtained from four farms/ranches, one in Monterey and three in San Benito Counties. Details of the results of the investigation of this outbreak have been reported [9].

An important tool in outbreak and environmental investigations is “fingerprinting” of strains to identify relatedness and/or potential sources of contamination. Pulsed-field gel electrophoresis (PFGE) has become the method of choice in these investigations due to the availability of the PulseNet database containing thousands of profiles submitted by public health labs for isolates associated with sporadic or outbreak illnesses [35]. Nevertheless, PFGE is a time- and labor-intensive method, requiring specially trained individuals to acquire, analyze and submit results comparable between multiple labs. A newer method, Multi-Locus Variable-number tandem repeat Analysis (MLVA), has been shown to be more reproducible than PFGE and better at discriminating between closely related EcO157 isolates [36]–[39], and is being evaluated by CDC as a potential next generation typing system. Discrimination of strain differences with MLVA data relies on hyper-mutable tandem repeat (TR) elements located at various places in the genome. Isolates that are closely related epidemiologically and/or by source (e.g. human clinical or food isolates from the same outbreak, animal host), often are identical or differ by only one or two TR changes at a single locus [36].

We report the results of surveillance studies of EcO157 incidence in multiple environments associated with EcO157 foodborne outbreaks. We report that surface water is a vehicle of transmission of EcO157 and a potential risk factor for pre-harvest contamination of leafy vegetables in the Salinas region. Our results demonstrate the suitability of MLVA for typing EcO157 strains isolated during complex environmental investigations. Furthermore, we report genotyping data measuring the relatedness of strains, useful for identifying potential environmental point sources of EcO157.

## Materials and Methods

### Sporadic and outbreak clinical isolates

Human clinical isolates were received from Dr. M. Janda at the California Department of Health Services (CDHS); S. Chu at the CDHS-LA County; Dr. K. Smith at the Minnesota Department of Public Health; D. Green at the Washington State Public Health Laboratory; C. Ball at the Idaho Bureau of Laboratories; W. Chmielecki at the Pennsylvania Department of Health; T. Monson at the Wisconsin State Laboratory of Hygiene; and the National Laboratory for Enteric Pathogens (NLEP), National Microbiology Laboratory (NML), Health Canada. Strains obtained from the DECA Reference Strain Set are available from the STEC Center, Michigan State University. A list of all strains used in this study, including source information, is provided in Supplemental Information, Table S1.

### Environmental sampling

Representative samples of soil, sediment, feces, and plants were collected from the environment into Whirl-Pak bags (Nasco, Modesto, CA) using either clean latex exam gloves or single-use sterile spatulas. Pig colon samples were collected by necropsy performed in the field (Jay MT, Cooley MB, Carychao D, Wiscomb GW, Sweitzer RA, et al. (2007) *Escherichia coli* O157:H7 in feral swine near spinach fields and cattle, central California coast).

**Figure 1:**
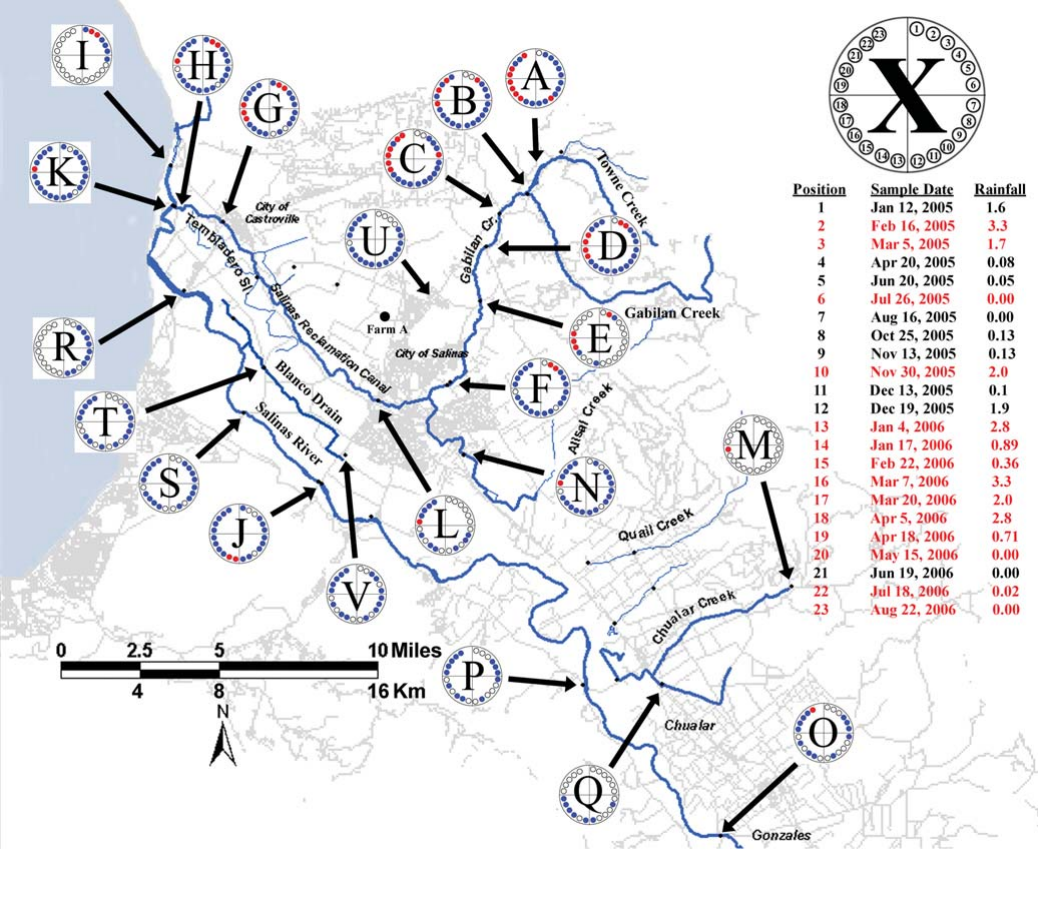
Map of Salinas, CA region showing sample locations. Farm A is marked as a black dot. The circle marked with “X” is coded by positions numbered clockwise 1 to 23 and representing the sampling dates show in the list below the circle. The approximate rainfall in cm for the 5 days prior to sampling is shown in the list. Dates indicated in red text indicate at least one EcO157 strain was isolated on that date. Circles on the map designated “A” through “V” correspond to the locations where samples were obtained; these are also listed in Table S1. Open circles within the “Location circle” designate that no sample was obtained on that date; blue circles designate that a sample was obtained, but no EcO157 strain was isolated; and red circles designate that a sample was obtained, and at least one strain of EcO157 was isolated.

Samples of water (100 ml) were collected into sterile, disposable bottles. The collection points were as close to the centers of a stream or pond as practical, and collection was accomplished by attaching bottles to a telescoping pole. GPS data and observations regarding appearance of the sample or the surroundings were recorded. Some samples were collected with cotton gauze swabs (“Moore swabs”) [40] by anchoring them in a water source to a monofilament line and retrieving them 5 days later [41]. The swabs were stored in marked Whirl-Pak bags. All samples were transported on ice. Sample locations in the Salinas watershed are shown in Figure 1. Additional details of watershed and 2006 spinach outbreak sample locations have been reported [9], [42], [43].

### Colilert testing

For watershed samples, quantification of total coliforms and *E. coli* was determined by the Colilert® QuantiTray 2000 method according to the manufacturer's recommendations (Idexx Laboratories, Westbrook, ME). Duplicate samples (111 ml) of surface water were collected from each site, transported to the laboratory on cold packs, and stored at 4°C overnight. The following morning, each sample was diluted 1∶10 and 1∶100 in Butterfield's phosphate buffer. Colilert® reagent containing 4-methylumbelliferyl-beta-D-glucuronide (MUG) was added to 100 ml of each dilution, and samples were inoculated into 97-well trays and sealed. After 24 hours of incubation at 35°C, wells were counted for yellow color from fermentation of O-nitrophenyl-beta-D-galactoside (ONPG), evidence of total coliforms, and blue fluorescence from MU (generic *E. coli*), and the Most Probable Number (MPN) were calculated from the manufacturer's data tables. Four control strains (*Pseudomonas aeruginosa*, ATCC 27853; *Klebsiella pneumoniae*, ATCC 13882; *E. coli*, ATCC 25922; EcO157, ATCC 43888) were cultured and tested simultaneously for each assay.

### Isolation method

Collected samples were transported and/or stored 12 to 24 hr on ice until the isolation method was initiated, usually the morning after collection. Our method was modified from a method described previously for isolation of EcO157 from cattle feces, carcass and hide samples [44]. Ten grams of sample, including soil, sediment, feces or tissue (plant or colon), were removed by hand using clean exam gloves or with a sterile spatula, and placed into a 250 ml sterile flask containing 90 ml tryptic soy broth (TSB; Becton Dickinson, Sparks, MD). Moore swabs were rinsed briefly in tap water to remove mud and sediment, and were added to sterile flasks (1 liter) containing 250 ml TSB for enrichment. The flasks were incubated for two hrs at 25°C with shaking at 200 RPM, then at 42°C for 8 hrs, and held at 4°C without shaking until the following morning. Water samples were enriched by adding 11 ml of 10× TSB (filter sterilized, 0.45 µm) to 100 ml of sample, and the mixture was incubated as described above.

One ml of the enrichment broth was tested by adding 20 µl of anti-O157 antibody bound to magnetic beads (Invitrogen/Dynal, Carlsbad, CA) and mixing them for 30 min. The beads were washed twice with PBS containing 0.05% Tween 20 (PBS-T), then they were re-suspended in 100 µl PBS [45]. Alternately, beads were incubated and wash using the Dynal BeadRetriever (Invitrogen/Dynal, Carlsbad, CA) using the EPEC/VTEC Protocol. With either method, 50 µl each of the resuspended beads were spread on Sorbitol MacConkey agar (Difco Labs; Detroit, MI) containing cefixime (0.05 µg/ml; Invitrogen/Dynal) and tellurite (2.5 µg/ml; Invitrogen/Dynal) (CT-SMAC), and on Rainbow Agar (Biolog, Hayward, CA) containing novobiocin (20 µg/ml; Sigma-Aldrich) and tellurite (0.8 µg/ml; Invitrogen/Dynal) (NT-Rainbow). To aid in identification of authentic EcO157 colonies, plates of both media were streaked with an EcO157 strain RM2315 marked with the Green Fluorescent Protein [46]. Plates were incubated at 37°C overnight (approximately 18 hr).

Suspect colonies and positive control EcO157 strain RM2315, were patched onto duplicate plates of Luria Broth (LB) agar (Fisher Scientific, PA) using a numbered grid and incubated at 37°C overnight. One set of the patched colonies was blotted onto Protran nitrocellulose membranes (BA 85, Whatman/Schleicher & Schuell; Sanford, ME). The membrane was washed with 25 mM TRIS pH 7.4, 0.15 M NaCl, 0.1% Tween 20 (TBS-Tween) and blocked by immersion in 10 ml of 0.5% casein, 0.01 M TRIS, 0.031 M sodium azide, 0.15 M NaCl pH 7.4 (blocker) with shaking for 30 minutes at RT. The membrane was incubated in anti-O157 IgG monoclonal antibody (MAb), 13B3 [47], diluted 1∶2000 in 10 ml 1× TBS-Tween containing 1% BSA, 0.02% KCl, 0.1% sodium azide (ELISA Diluting Buffer) and with shaking for 30 minutes at RT. The membrane was washed four times with 100 ml TBS-Tween, then incubated in goat anti-mouse IgG conjugated with alkaline phosphatase (Invitrogen/Zymed, Carlsbad, CA) diluted 1∶2000 in ELISA Diluting Buffer for 30 minutes with shaking at RT. The membrane was washed four times with TBS-Tween, then twice in distilled water. The membrane was developed by the addition of 10 ml of a 0.15 mg/ml and 0.3 mg/ml solution of 5-Bromo-4-Chloro-3-Indolyl Phosphate (BCIP) and Nitro Blue Tetrazolium (NBT) Sigma-Aldrich, B5655), respectively. During multiple isolation procedures, we noted that authentic EcO157 colonies were distinguishable from apparent antibody-positive non-EcO157 bacteria by the rate of the color reaction compared to EcO157 strain RM2315.

### Virulence gene detection via PCR

Putative EcO157 colonies identified by colony blots as described above were analyzed further by real-time PCR for the presence of the *rfb*E gene. Primers were designed based on a 497 bp region of the *rfbE* gene reported to be specific for EcO157 [48]. The 20 µl reaction mix included 10 µl Stratagene Brilliant QPCR master mix, 0.3 µM each *rfb*E primers (5′-TTTCACACTTATTGGATGGTCTCA3′ and 5′ TGAGTTTATCTGCAAGGTGATTCC-3′), and 0.1 µM of the probe (5′ 6-FAM-TTCTAACTAGGACCGCAGAGGAAAGAGAGGAATTA-BHQ-1-3′, Biosearch Technologies, Inc., San Francisco, CA). Bacteria were transferred from the duplicate patch plate directly into the PCR tube using a sterile toothpick. Control tubes containing no template, or EcO157 strain RM2315 bacteria, were included for amplification in a Stratagene MX3000P Real-Time PCR machine (95°C for 5 min, then 60 cycles of 95°C for 15 sec and 60°C for 45 sec).

Colonies producing a positive reaction for the *rfb*E gene were streaked from the duplicate patch plate for isolation of single colonies and were analyzed further for the *fli*C (H7) gene using the method of Fratamico et. al. [49]. Each 25 µl reaction contained 1× ThermoPol buffer (New England Biolabs, Ipswich, MA), 200 µM each dNTP, 0.25 µM each primer, 2.5 U TAQ, and 1 µl template. Thermal cycling parameters were 95°C for 5 min, then 30 cycles of 94°C for 30 sec, 58°C for 30 sec, 72°C for 30 sec, and a final extension at 72°C for 5 min. DNA template was prepared by re-suspending approximately 1 µl of cells scraped from a plate with a 1 µl sterile loop into 100 µl of distilled water with subsequent boiling for 20 minutes. Debris from boiled cells was removed by centrifugation at 5000 RPM for 10 min.

Other virulence genes (*stx*1, *stx*2, *eae*, *hly*) were detected by a multiplex reaction described previously [50]. Each 15 µl reaction contained 1× Qiagen multiplex PCR master mix (Qiagen, Valencia, CA; #206143), 0.25 µM each primer, and 1 µl of template. Thermal cycling parameters were 95°C for 15 min, then 35 cycles of 95°C for 1 min, 65°C for 2 min, and 72°C for 1.5 min. Annealing temperatures decrements from 65°C to 60°C between cycles 10 and 15, and elongation time increments from 1.5 min to 2.5 min between cycles 25 and 35. All PCR ingredients were purchased from New England Biolabs. Reactions were done on a Tetrad thermal cycler (Bio-Rad/MJ Research, Hercules, CA) and run on a 2% agarose gel.

### PFGE method

Selected isolates were typed using the standard PulseNet procedure with both *Xba*I and *Bln*I restriction enzymes [35]. Dendrograms for comparing the isolates were constructed by analysis of the profiles for both digests with bandmatching and phylogenetic clustering analysis methods of Bionumerics (Applied Maths, Sint-Martens-Latem, Belgium). PFGE profiles of the isolates were compared against those present in the PulseNet database.

### MLVA method

MLVA was performed using capillary electrophoresis methods described previously [37], [39]. Essentially, 10 loci are amplified in three multiplex PCR reactions using fluorescent primers. Reaction 1 contained primers for VIC-Vhec1, NED-Vhec3, FAM-Vhec4, FAM-Vhec5; reaction 2 contained primers for VIC-Vhec1, NED-Vhec2, VIC-Vhec6, FAM-Vhec7 [37]; and reaction 3 contained primers for FAM-O157-17, NED-O157-19, VIC-O157-37 [39]. All fluorescent primers were obtained from Applied Biosystems (ABI, Foster City, CA). Each PCR reaction of 10 µl contained 1× multiplex PCR master mix (Qiagen), 0.2 µM of each primer and 1 µl template. Thermal cycling parameters for reactions 1 and 2 were 95°C for 15 min, then 25 cycles of 94°C for 30 sec, 63°C for 90 sec, 72°C for 90 sec, with a final extension at 72°C for 10 min. Parameters for reaction 3 were 95°C for 15 min, then 35 cycles of 94°C for 20 sec, 65°C for 20 sec, and 72°C for 20 sec, with a final extension of 72°C for 5 min. The multiplex reactions were pooled and diluted 1∶50 into distilled water. One µl of this dilution was added to a mixture of 12 µl HiDi formamide (ABI) and 0.08 µl of a MW standard ladder (ROX-labeled MapMarker 1000, Bioventures, Inc., Murfreesboro, TN), and the mixture was heat-denatured for 5 min at 95°C, cooled on ice for 2 min, loaded onto the ABI 3130xl Genetic Analyzer, and run for 2100 seconds using the default settings for fragment analysis with “Dye Set D” (ABI) in a 50 cm array containing POP7 polymer. The sizes of fragments were determined using GeneMapper software (ABI). If more than one fragment was detected for a single locus, the fragment with the highest level of fluorescence was selected for TR determination. Fragment size was converted to number of TR by subtraction of the amplified, non-repeat sequences and division by the repeat size. Fractional repeat numbers were rounded to the nearest whole repeat number for purposes of comparison. MLVA genotypes for each isolate tested in this study are listed in Supplemental Information, Table S1.

### Phylogenic, statistical methods and precipitation data

A minimal spanning tree of the MLVA types was created using the MST algorithm in BioNumerics regarding TRs as categorical coefficients. All coefficients were equally weighted. When solutions with identical calculated distances were obtained, a priority rule was applied for linkage in the following order; the highest number of single-locus variants, the highest number of double-locus variants and the highest number of entries. Clusters were selected based on criteria that neighbors within the cluster must be single locus variants and the resulting cluster must contain at least 3 MLVA types. The allelic diversity was based on Nei′s diversity [51], which is the calculation 1-Σ (allele frequency)^2^. Wilcoxon signed rank test or t test was used for pairwise comparisons between generic *E. coli* levels and EcO157 incidence (SigmaStat 3.0, SPSS, Inc., Chicago). Precipitation totals were determined from the average of daily accumulative data collected at four weather sites as part of the California Irrigation Management Information System (CIMIS). The sites included data for northern Salinas, southern Salinas, Castroville and the Salinas airport on the collection date and four days prior to the collection date. Flow rates in Gabilan Creek, Salinas River and Reclamation Canal were available at USGS National Water Information System (http://waterdata/usgs.gov/ca).

## Results

### Sampling of a farm associated by traceback with three separate outbreaks in 2002–2003

From June through December 2004, 178 samples were collected from a farm (Farm A, Figure 1) that was identified during traceback investigations as having provided produce associated with three separate outbreaks (Spokane, WA 2002, San Diego, CA 2003, and San Mateo, CA 2003). Samples included soil, water, compost, wild animal feces, sediment and plants and were processed for isolation of EcO157. Further details of the investigation are published in a CDHS report [5]. The only sample yielding EcO157 was a creek sediment sample collected adjacent to Farm A in July 2004. A single strain of EcO157 (Table S1, RM4403) was isolated from the sample. Strain RM4403 was *fli*C (H7)-positive by PCR; virulence genes *stx*1, *stx*2, *ea*e and *hly* also were positive by PCR (data not shown). However, MLVA and two-enzyme PFGE (*Xba*I and *Bln*I) indicated that RM4403 did not match closely any of the clinical isolates from the three associated outbreaks (Table S1: see “Source” OB6, OB7, OB8; data not shown).

Potential upstream sources of EcO157 surveyed subsequently included a goat ranch and horse ranch, both located<2.4 km upstream. The goat ranch, situated on a hill approximately 800 meters from the creek, contained approximately 40 goats, 6 horses/ponies, and, infrequently, a few cows. Drainage from this property fed into a tributary of the creek. The horse ranch, situated on the creek approximately 1.6 km upstream, contained 12 horses that had direct access to the creek. Nevertheless, water and sediment samples taken immediately downstream of these properties were consistently negative for EcO157.

### Sampling of the Salinas Valley watershed, 2005

Several other produce-related outbreaks have been linked to the Salinas region, some of which may have been due to pre-harvest contamination. The California Central Coast Regional Water Quality Control Board (CCRWQCB) reported levels of generic *E. coli* above acceptable levels at several locations in the watershed [52]. Farm A, which floods almost every winter, would be exposed potentially to these contaminated waters. Indeed, surface water routinely floods onto fields elsewhere in the Salinas region. Therefore, selected sites in the Salinas Valley watershed were surveyed for the presence of EcO157 as a measure of the potential contribution to pre-harvest contamination. We initiated a comprehensive investigation of the Salinas watershed in January 2005, collecting 584 water samples from 22 watershed locations (Figure 1). Samples were collected in collaboration with the CCRWQCB as part of an on-going investigation. Sample locations and sample frequency were selected in coordination with the CCRWQCB and the California Department of Health Services. Due to the potential influence of flooding on pre-harvest contamination, additional samples were collected after significant rainfall during winter months (November to April).

At least one strain of EcO157 was isolated from 38 (6.5%) of the total water samples collected. Two-hundred and thirteen Moore swab samples were placed at several of the same locations starting in December 2005; 37 were positive for EcO157 (17.4%). A combined total of 75 of the water “grab” and Moore swab samples were positive during the 19 month watershed survey (12.8%).

**Figure 2:**
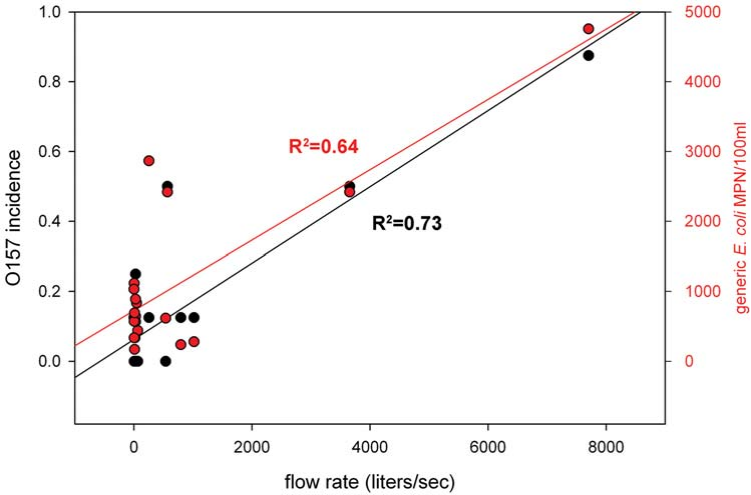
Correlation of water flow, the concentration of generic *E. coli*, and the incidence of EcO157 in the Gabilan Creek (Salinas, CA). Linear regression R^2^ values are indicated. EcO157 incidence is calculated as the fraction of the number of positive samples compared to the total samples obtained. The flow rate was obtained from data from the USGS sampling station #11152600 located at position D (see Figure 1). EcO157 incidence and generic *E. coli* concentration were determined with duplicate samples.

**Figure 3:**
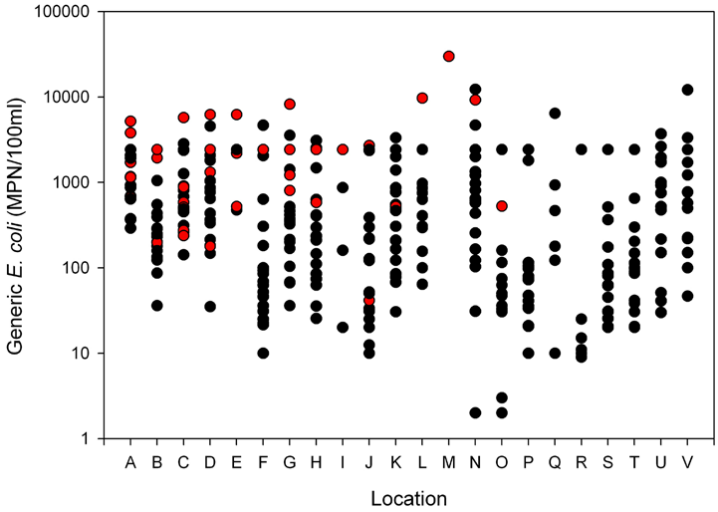
Concentration of generic *E. coli* in the Salinas watershed. Sample locations refer to Figure 1. Red and black dots indicate isolation or non-isolation of EcO157 for the corresponding duplicate water samples, respectively.

The majority of the EcO157 positive samples (42 of 75 = 56%) were collected at sites in the Gabilan Creek (designated A to D in Figure 1), especially upstream sites closer to grazing. The concentration of generic *E. coli* determined for duplicate water samples collected from these locations varied extensively, with the highest concentrations occurring during months with the highest measured water flow (Figure 2). During these months, the flow in the Gabilan Creek at elevated areas can increase more than 1000-fold over base flow, resulting frequently in flooding downstream (data from water flow meter at location D, “Gabilan Creek”, see [53]). The average generic *E. coli* levels measured for locations A to D increased by five-fold during these months, and the incidence of EcO157 increased from undetectable to a high of more than 85% of the samples being positive. Throughout the watershed, high concentrations of generic *E. coli* coincided generally with a high incidence of EcO157 (P = 0.001, Figure 3). Nevertheless, generic *E. coli* levels did not correlate well with the incidence of EcO157 when individual sampling sites were compared, except location G (P = 0.009). Generic *E. coli* concentrations were frequently high at sampling times when EcO157 was not recovered.

### Spinach outbreak investigation, 2006

**Figure 4:**
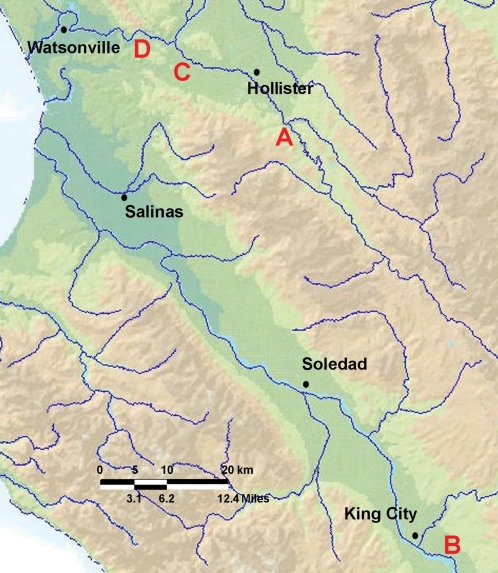
Map of Salinas and San Juan valleys showing the positions (A–D) of F/Rs implicated in the traceback investigations associated with the baby spinach outbreak 2006. Blue lines on the map are waterways.

During this 19-month study of the Salinas Valley watershed, a large, multi-state outbreak occurred with illnesses in August/September 2006 linked to baby spinach produced in Monterey and/or San Benito counties. USDA-ARS laboratories processed 222 environmental samples collected at four farms/ranches (F/R) identified in the traceback investigation (Figure 4, F/R A, B, C, D). Water at each of the four ranches was sampled and tested due to the incidence and probable transport of EcO157 shown in the Salinas watershed. Surface water was located in close proximity (<0.8 km) to fields on all four ranches. However, unlike Farm A noted previously, the spinach outbreak-investigated fields (F/R A, B, C, D) were not subject to routine flooding. Thirty-two water samples were collected, including well water used for irrigation and surface water (holding ponds and nearby streams). Water sample locations were selected based on their proximity either to fields or cattle. Other samples were collected to investigate other potential sources of contamination included feces (cattle, feral pig, deer), soil and sediment. Fenced cattle were located directly adjacent to fields at F/R A and 2.2 km or 0.6 km from fields at F/R B and C, respectively. Cattle were not evident at or near F/R D. From 222 samples, 27 were positive for EcO157, which included 21 positive samples at F/R A and 6 positive samples at F/R B. Positive samples obtained at F/R A came from water, sediment, cattle and pig feces, and dust. Water and sediment samples that were positive for EcO157 were all collected at a location on F/R A near cattle and approximately 1.5 km from the fields. All EcO157-positive samples from F/R B were from cattle feces.

### Genotypic comparison of EcO157 isolates

There were 102 samples analyzed during the combined watershed and outbreak studies that were positive for EcO157. Multiple isolates were subcultured from one or both of the two different selective agar plates for each sample. then confirmed as EcO157 by anti-O157 MAb and *rfb*E analyses, and genotyping by MLVA. MLVA resulted in identification of 92 different MLVA types for the 1,301 isolates analyzed (Table S1). Isolates confirmed as EcO157 were analyzed subsequently by PCR for the presence of *fli*C (specific for H7 antigen) and four putative virulence genes. All of the EcO157 isolates found in this study contained *fli*C (flagellin gene), *eae* (intimin), and *hly* (hemolysin gene). It is noteworthy that all of the isolates described in this study were also positive for *stx*2, with the exception of one isolate that was also negative for *stx*1 (Table S1, MLVA 7). Thirty-eight other MLVA types were negative for *stx*1 (Table S1). Isolates of the same MLVA type always had the same profile of virulence genes by PCR analysis.

**Figure 5:**
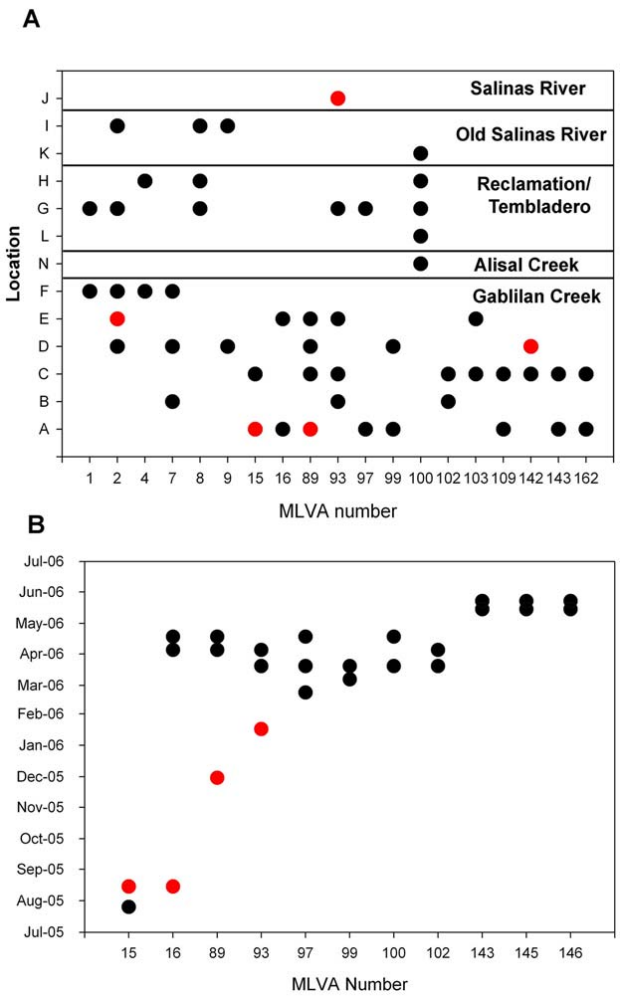
MLVA types compared to (A) Salinas watershed location or (B) sample date. Location designations refer to Figure 1 and are separated by horizontal lines in (A) to indicate separate waterways. Black and red dots indicate isolation of the MLVA type at the indicated location or date. Red dots indicate that differences were detected between that isolate and the other isolates of that MLVA type by PFGE.

Interestingly, most MLVA types were isolated only once during the watershed and outbreak investigations (61 of 92 types). However, several MLVA types were isolated multiple times from samples obtained at different times or locations. For example, there were 19 cases where the same MLVA type was isolated at more than one sample location during the watershed survey (Figure 5A). Most (11 of 19) of the multi-site MLVA types were isolated at multiple locations on the Gabilan Creek, but not at other watershed locations. MLVA type 8 strains were isolated only from multiple locations in the Tembladero Slough and Old Salinas River, but were not isolated from upstream sites. It is noteworthy that occasionally, the same MLVA type was isolated on the same day from locations separated by relatively large distances, but in the same drainage path. This occurred on three separate sampling dates. MLVA types 1, 2 and 4 strains were recovered repeatedly on Feb. 16, 2005 from multiple sites along the Gabilan watershed and the Tembladero Slough (Figure 5, Table S1). An MLVA type 2 strain was also recovered from a site on the Old Salinas River on that same day. The total distance via the waterways from the Gabilan sample site (D) to the Old Salinas River site (I) is approximately 30 km (Figure 1). Similarly, MLVA type 9 strains were recovered from sites along both the Gabilan and the Old Salinas River on March 23, 2005, and MLVA type 100 strains were recovered from sites on the Alisal Creek, Reclamation Ditch and Tembladero Slough (maximum separation of 25 km) on April 18, 2006 (Figure 1). It is important to note these three sample dates were during the winter and that flow in the Gabilan and Reclamation Ditch was high (568, 852 and 624 liter/s on the respective sample dates).

Additionally, there were 10 cases where the same MLVA type was isolated from samples collected on different dates (Figure 5B); on 8 occasions, the same MLVA type was isolated from samples collected on subsequent dates at the same location. In contrast, MLVA 97 strains were isolated from Tembladero Slough nearly two months after its initial isolation from the Gabilan. Additionally, MLVA 93 strains were isolated initially from the Salinas River and again, two months later, from both the Gabilan and Tembladero Slough.

EcO157 was isolated also from a single sample of cattle feces collected near location A. This sample yielded two MLVA types (15 and 16), both of which were isolated also from water samples collected from the Gabilan Creek (Figure 5B). The MLVA 15 strain was isolated from a water sample collected at location C three weeks prior to the feces sample collection. The MLVA 16 strain was isolated from water samples collected nearly eight months later at location A and E.

### Incidence of EcO157 at an elevated site near a potential point source

EcO157 was isolated most frequently (22% of all positive samples) at sample location A (Figure 1), and upstream of location A on Towne Creek (a tributary to Gabilan Creek). Several potential sources of contamination were evident. For example, at one site a small tributary moved through a corral containing approximately eight head of cattle. Soil samples taken near the creek inside the corral were positive for EcO157 (Table S1: MLVA types 143 and 145). No other sources of contamination were evident up to 0.8 km downstream, suggesting these cattle as an isolated point source.

**Figure 6:**
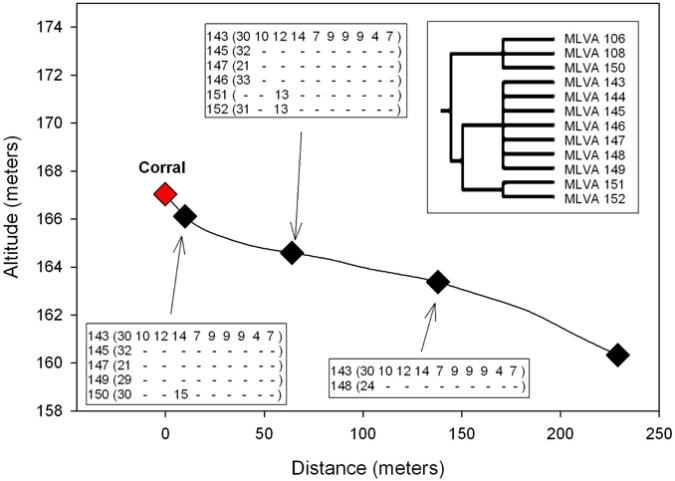
The persistence and transport of EcO157 in a stream in an elevated area near a point source. The change in stream elevation is indicated as a function of the distance from a corral with cattle. Sampling positions are indicated by: ⧫; different MLVA types isolated from each location are indicated. The inset shows a phylogenetic tree designating the relationship among the MLVA types isolated in this study (see Figure 7 and Table S1 for more detail).

To determine whether the incidence of EcO157 correlated spatially with this potential point source, four duplicate water samples were collected at each of seven locations, at distances from 10 to 730 meters downstream from the corral. In the five days prior to sampling, the total rainfall was 0.8 cm and water flow was estimated at 0.1–0.2 m/s depending on location. Isolates recovered from the corral were of MLVA types indistinguishable or similar to those recovered from downstream water samples (Figure 6), such as MLVA types 143 and 145 strains isolated from all samples collected at 10 and 65 meters downstream, and MLVA 143 isolated from two of four samples collected at 135 meters downstream. None of the samples collected at 230 meters and further from the point source were positive for EcO157(Figure 6). Although isolates representing multiple different MLVA types were obtained from these sites, the isolates appeared to be related, based on subtle differences in the number of TR at Vhecs 1, 3 and 4 loci (see inset for Figure 6).

### Clustering of MLVA types

**Figure 7:**
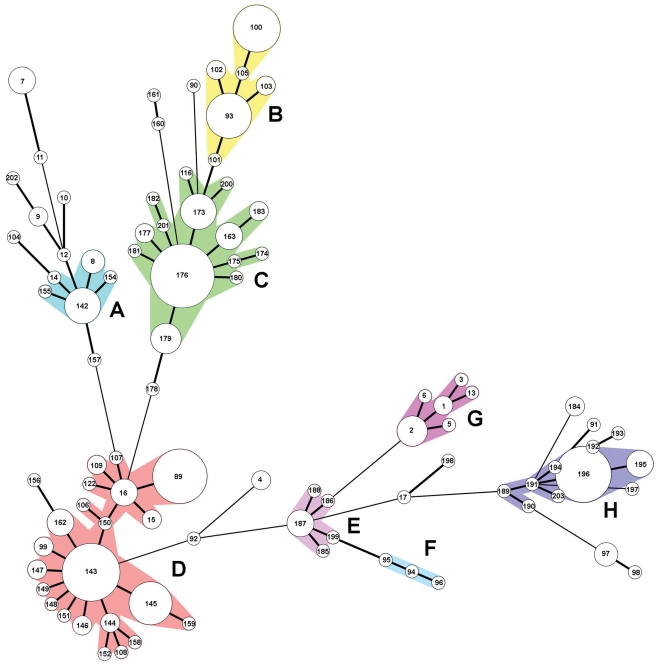
A comparison of the phylogenetic relationships among 92 MLVA types. The size of the circles indicate relative number of isolates of each MLVA type (numbers in the circles) recovered from the environment. Those MLVA types shown with the smallest circles were isolated only once. Letters A–H indicate clusters (colored regions) in which types differ at only 1 of the 10 MLVA loci from a neighbor.

Phylogenetic analyses indicated that 70% of the environmental isolates clustered into eight groups (labeled as A–H in Figure 7). A cluster was defined as those MLVA types that differ at only a single locus from its nearest neighbor. Also, the strains within any of the eight clusters had the same *stx*1 gene profile. For example, strains in Clusters A, D, E, F and G all contain *stx1*, whereas Clusters B, C and H do not. Furthermore, the strains represented within Clusters C, D, E and F were related to one another also spatially, i.e., they were isolated from samples obtained within 8 km of one another. For example, strains in Clusters D were isolated exclusively from the Gabilan Creek and its tributaries. Cluster F strains were isolated from Salinas River samples only. Strains in clusters C and E were isolated only from F/R A and B, respectively. In contrast, the remaining four clusters (A, B, G and H) contain strains isolated from divergent locations. Clusters A and G contain strains from both the Gabilan and the Tembladero Slough, perhaps reflecting that they are connected hydrologically (Figure 1). However, Cluster B contains strains from several locations in the Salinas watershed, some of which are not connected hydrologically (e.g., locations C, J and N). Similarly, Cluster H contains strains from both F/R A and B, which have no watershed connection (Figure 4).

### Comparison of MLVA and PFGE data for EcO157 strains

**Figure 8:**
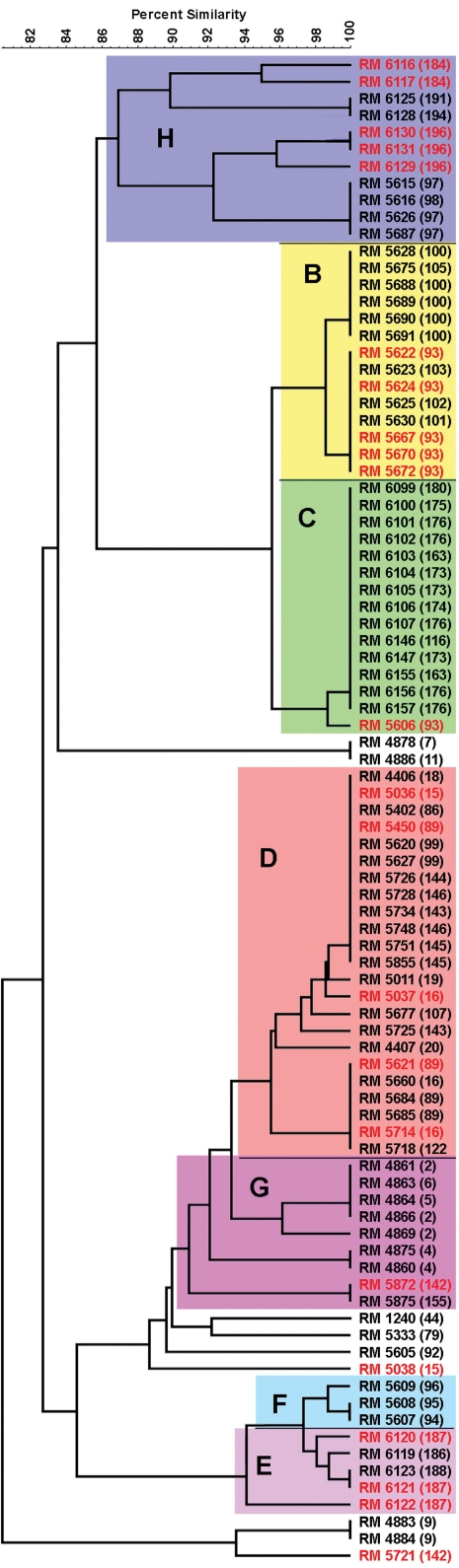
Dendrogram of selected isolates from PFGE data. Taxa are designated as RMXXXX (##), where RMXXXX is the isolate name and ## is an arbitrary number assigned to that MLVA type [refer to Table S1]. Taxa in red type are identical MLVA types differentiated by PFGE. Colored clusters (labeled B–H) are similar to labeled MLVA clusters in Figure 7.

The relationships between selected sets of strains were investigated further by PFGE analysis using two enzymes *Xba*I and *Bln*I. Strains for comparison were selected primarily to further characterize strains of identical MLVA types that had been isolated from different locations and/or at different times. Additional strains were included in the PFGE analysis to enhance the comparisons of closely related MLVA types collected during the 2006 baby spinach outbreak investigation. In many cases, PFGE data failed to discriminate strains of closely related MLVA types (Figure 8). For example, several isolates in MLVA clusters B, C and D were indistinguishable by PFGE. Nevertheless, PFGE analysis distinguished occasionally between identical MLVA types (Figures 5 and 7). Some of these differences in PFGE patterns contrasted with the hypothetical transport processes of EcO157, as interpreted from the MLVA data. For instance, an MLVA type 16 strain was isolated from a water sample collected eight months after it was isolated from a cattle fecal sample. However, PFGE data indicated the MLVA type 16 strains isolated from feces and from water were different (Figure 8, cluster D). Similarly, MLVA 93 type strains were isolated from both the Gabilan and the Salinas River, sites having no hydrologic connection. However, PFGE data distinguished between the MLVA 93 strains isolated from the Salinas River and the multiple MLVA 93 strains from the Gabilan watershed (Figure 8, clusters B and C). PFGE data were also inconsistent with MLVA data by distinguishing between identical MLVA types 15, 89 and 142 from the Salinas watershed, and also some strains isolated from various samples collected during the spinach outbreak investigation. For example, MLVA 196 isolates from F/R A pig feces and sediment samples were distinguishable by PFGE (Figure 8, cluster H). Similarly, MLVA 184 and 187 isolates from different cattle feces from F/R B were distinguishable by PFGE (Figure 8, clusters E, H).

### Comparison of environmental and human clinical EcO157 strains by MLVA

A set of 105 human clinical strains of EcO157, associated predominately with sporadic illnesses in California, and outbreak strains from other states, was typed by MLVA for comparison to MLVA data of environmental isolates. Strains from outbreaks associated with apple juice (1996), taco meat (1999), spinach (2003) and lettuce (1999, 2002, 2003, 2005), were included in this comparison (Table S1). Comparison of the MLVA data shown in Table S1 revealed a number of strains linked by identical or very similar MLVA types, but without an epidemiological or spatial connection: (a) two environmental isolates from F/R A (RM6103, RM6155) obtained during the 2006 spinach outbreak investigation were exact matches to the major MLVA type of>40 clinical isolates associated with the 2006 spinach outbreak (MLVA 163); (b) an environmental isolate from feral swine feces collected at F/R A (RM6146) had an MLVA type identical to a clinical strain from a sporadic illness occurring in California in 2006 (RM5658, MLVA type 116, Table S1); and (c) two clinical strains from sporadic illnesses in California (MLVA types 60 and 86) were related by MLVA to environmental isolates (Table S1). MLVA type 86 strains differed from environmental isolates within Cluster D by two to seven TR at Vhec1. MLVA type 60 varied from MLVA types of other isolates in Cluster B by one to four TR at Vhec1 and one to three TR at Vhec4 (Table S1). Clinical isolates typed as MLVA types 112 and 129 were not associated with any MLVA clusters, but rather to individual environmental isolates of MLVA types 90 and 4, respectively.

### Comparison of environmental EcO157 strain PFGE profiles in CDC PulseNet

PFGE patterns determined for selected environmental strains were compared against the CDC PulseNet database, which contained during the preparation of this study, *Xba*I patterns for>22,000 EcO157 isolates (Table 1). Numerous environmental isolates from this study matched *Xba*I patterns present in the PulseNet database (17 of 29 strains analyzed). Ten of the 17 PFGE profiles matching PulseNet *Xba*I patterns, also matched *Bln*I patterns. Thirteen MLVA types identified for environmental isolates corresponded to 10 different PFGE profiles (*Xba*I and *Bln*I) associated in PulseNet with 10 separate outbreaks; 8 of the outbreaks involved multiple states (Table 1: “PulseNet Cluster(s)”). For example, multiple MLVA types were identified for EcO157 watershed strains with *Xba*I-*Bln*I profiles EXHX01.0343-EXHA26.0569 (MLVA types 89, 99), EXHX01.0200-EXHA26.0015 (MLVA types 101, 102, 103), and EXHX01.3099-EXHA26.0265 (MLVA types 94, 95, 96). The MLVA types corresponding to the EXHX01.3099-EXHA26.0265, EXHX01.0343-EXHA26.0569 and EXHX01.0200-EXHA26.0015 profiles were closely related based on 8/10, 8/10 and 7/10 MLVA loci, respectively. An example of the value of MLVA can be discerned from a comparison of strains with the *Xba*I-*Bln*I profile EXHX01.0047-EXHA26.0015, a common two-enzyme profile represented by>6% of the submitted profiles in the PulseNet database. MLVA data were available for some clinical isolates designated with this common profile and also for two strains reported previously in a comparison of PFGE and MLVA [39] (Table 2). MLVA of this limited set of strains with no apparent linkage epidemiologically (i.e. not outbreak related) revealed that these “EXHX01.0047-EXHA26.0015” strains in the PulseNet database are not related closely. Indeed, the closest similarity was with only 6 of 10 MLVA loci with identical number of TRs.

**Table 1:** Comparison of MLVA types of *Ec*O157 environmental strains with PFGE profile designations.

MLVA type^a^	Strain no.^a^	PulseNet *Xba*I^b^	*Xba*I, % of PulseNet^c^	PulseNet *Bln*I^d^	PulseNet Cluster(s)^e^
1	RM4859	EXHX01.3536	0.004	EXHA26.1411	
2	RM4869	EXHX01.3578	0.004	EXHA26.1411	
3	RM4862	EXHX01.0221	0.031	EXHA26.1411	
13	RM4888	EXHX01.3537	0.004	EXHA26.1411	
15	RM5036	EXHX01.1271	0.366	EXHA26.0569	CA:Oct-03, Multi:Oct-04
15	RM5038^f^	EXHX01.0263	0.374	EXHA26.0257	
16	RM5037^f^	EXHX01.0202	0.113	EXHA26.0569	Multi:Jun-05, Nov-05
17	RM4403	EXHX01.0122	0.053	EXHA26.0354	
89	RM5450	EXHX01.0343	0.167	EXHA26.0569	Multi:Sep-04
89	RM5621	EXHX01.3535	0.070	EXHA26.0569^h^	
90	RM5603	EXHX01.2216EXHX01.2216	0.070	EXHA26.0684	NC:Nov-04
91	RM5604	EXHX01.0509	0.031	EXHA26.1412	
92	RM5605	EXHX01.3455	0.009	EXHA26.0569	
93	RM5606	EXHX01.0047	6.112^g^	EXHA26.0015	Multi state, Mar-03-Aug-06
94	RM5607	EXHX01.3099	0.048	EXHA26.0265	CA:May-03
95	RM5608	EXHX01.3099	0.048	EXHA26.0265	CA:May-03
96	RM5609	EXHX01.3099	0.048	EXHA26.0265^h^	CA:May-03
97	RM5615	EXHX01.2221	0.044	EXHA26.1395	
98	RM5616	EXHX01.2221	0.044	EXHA26.1395	
99	RM5620	EXHX01.0343	0.167	EXHA26.0569	Multi:Sep-04
100	RM5628	EXHX01.0200	0.959	EXHA26.0332	
101	RM5630	EXHX01.0200	0.959	EXHA26.0015	Multi:Aug-05, Jun-06
102	RM5625	EXHX01.0200	0.959	EXHA26.0015	Multi:Aug-05, Jun-06
102	RM5673	EXHX01.0200	0.959	EXHA26.0015	Multi:Aug-05, Jun-06
103	RM5623	EXHX01.0200	0.959	EXHA26.0015	Multi:Aug-05, Jun-06
104	RM5674	EXHX01.1031	0.194	EXHA26.0982	Multi:Jun-06
105	RM5675	EXHX01.0200	0.959	EXHA26.0332	
106	RM5676	EXHX01.3535	0.070	EXHA26.0569^h^	
107	RM5677	EXHX01.2838	0.070	EXHA26.0569 h	
108	RM5678	EXHX01.3535	0.070	EXHA26.0569 h	
109	RM5679	EXHX01.3535	0.070	EXHA26.0569 h	
122	RM5718	EXHX01.3535	0.070	EXHA26.0569 h	
163^i^	RM6103	EXHX01.0124	?	EXHA26.0015	Multi:Aug/Sep-06

a See Table S1 for MLVA data and source information.

b *XbaI* profile in PulseNet database (last checked Dec, 2006) matching designated strain. *Xba*I patterns EXHX01.3535, EXHX01.3536, EXHX01.3537 were new to the PulseNet database at the time of submission.

c Frequency of this *XbaI* profile in PulseNet database.

d *BlnI* profile in PulseNet database (last checked Dec, 2006) matching designated strain. *Bln*I patterns EXHA26.1395, EXHA26.1411, EXHA26.1412 are new to the PulseNet database.

e Clusters of illnesses identified from PFGE profiles submitted to PulseNet as *XbaI* profile, or both *XbaI* and *BlnI* profiles. Designation is by state (2 letter abbreviation):month-2 digit year; Multi, multiple states.

f RM5038 and RM5037 were isolated from the same sample of feces; MLVAs 15 and 16 differ by one TR only in locus 4.

g *XbaI*-*BlnI* profile EXHX01.0047-EXHA26.0015 represents>6% of the PulseNet database (see Table 2).

h One band difference between the profile for this strain and the *BlnI* PulseNet profile designated.

I Major MLVA type for human clinical strains associated with the spinach outbreak.

**Table 2:** MLVA differences between strains of the common PulseNet PFGE profile, EXHX01.0047-EXHA26.0015^a^.

Name	Source^b^	Approx. isolation date	MLVA genotype^c^
RM5652	Human (CA)	Feb-06	15-9-11-18-7-9-9-4-7-6
RM5606	Swab (CA)	Jan-06	20-9-11-14-7-9-8-4-7-6
RM5187	Human (CA)	Sep-05	20-9-16-20-7-9-4-4-7-6
RM5329	Human (CA)	Jul-05	20-8-14-16-7-9-8-4-7-8
RM6051	Human (PA)	Sep-05	20-9-13-15-7-9-8-4-7-7
VA_01-577^c^ ^d^	Human (VA)	2001	29-7-7-15-X-X-8-4-7-7
F7410^d^	Human (IN)	2000	32-7-13-15-X-X-9-4-7-8
RM6053	Human (PA)	Aug-06	38-9-14-18-7-9-10-4-7-7

a PulseNet patterns designated as described previously [35].

b Source of isolate (state).

c The number of TR are listed in the order noted in Table S1.

d Data for strains designated VA_01-577 and F7410 are from a previous study comparing PFGE and MLVA data [39]. X, not determined for these two strains.

## Discussion

We have described the incidence of EcO157 in an important leafy vegetable production region. Some molecular similarities among environmental strains raise questions regarding potential spatial connections and persistence of certain strains in the environment. These studies were stimulated by the isolation of a single strain of EcO157 from the sediment in a creek adjacent to a farm identified in three separate traceback investigations (Table S1, RM4403). However, the strain was different by PFGE and MLVA from clinical strains associated with the three outbreaks (Table S1, see “Source”: OB6, OB7, OB8). This result only indicated that EcO157 was in the production environment as a potential source of contamination, but no definitive mechanism for pre-harvest contamination could be discerned. The potential for produce contamination associated with the watershed was investigated further by assessing the incidence, survival and transport of EcO157, initially in the Salinas watershed, and subsequently on farms and ranches implicated by traceback investigation to the spinach outbreak. The results obtained from both the Salinas watershed study and the spinach outbreak investigation indicated that EcO157 was isolated more frequently from samples obtained near or on grazing land, compared to other locations. These results are consistent with reports of frequent incidence of EcO157 in numerous surveys of cattle in other locations of the country [54]–[62] and incidence in water [63]–[66].

The incidence of EcO157 was not uniform in the watershed. EcO157 was frequently isolated from elevated regions of Gabilan Creek, but much less frequently elsewhere. This would possibly explain why EcO157 was only found once in 178 samples from Farm A. Frequent isolation from the Gabilan watershed suggests there may be a higher concentration of EcO157 in this region compared to other sites. However, 45% of duplicate samples collected 30 seconds apart produced inconsistent results, i.e., EcO157 was isolated from one sample, but not the other, suggesting that the incidence and concentration of EcO157 in the watershed are dynamic events. Alternatively, the incidence of EcO157 may be uniform, but present at low concentrations. During the rainy season, it is anticipated that large volumes of run-off water would collect, dilute and disseminate pathogens from point sources (e.g. animal feces containing high concentrations), but resulting in concentrations close to or below the level of sensitivity of our method of detection of EcO157. Our isolation method was adapted from a sensitive EcO157 isolation procedure developed for cattle feces and hides [67]. However, water, wildlife feces, soil and other environmental samples often yielded growth of many non-target bacterial CFU on the selection plates that interfered with identification and selection of authentic EcO157 CFU. Background flora can decrease the sensitivity of any isolation method, thus resulting in an apparent incidence of EcO157 lower than may exist. Moore swabs for 5-day sampling improved the recovery of EcO157, but they also do not support quantification of viable EcO157, since enrichment is required. Research is ongoing to improve the sensitivity and speed of methods for isolating both EcO157 and *stx*-positive non-O157 *E. coli* from a variety of samples.

### Rainfall and incidence of EcO157

The 19-month period of sampling of the watershed incorporated two rainy seasons, facilitating measurement of a correlation between incidence of EcO157 and stream flow (Figure 2). This correlation could only be shown in the Gabilan where the incidence is highest. Nevertheless, even in the Gabilan Creek, the correlation was not very tight (R^2^ = 0.73), primarily due to very few occurrences of high EcO157 incidence. It is not known if rainfall (and subsequent flooding) releases bacteria already existing in the creek sediments, or if it washes contamination into the creek by run-off, although it is probable that both events occur. Studies with laboratory models indicate that EcO157 is attached predominantly to very small particles (2 µm) [68], [69]. These particles are too small to settle, minimizing interactions with the sediment and restricting the bulk of the contamination from entering the sediment. However, a process called hyporheic exchange can lead to high rates of suspended particle deposition in sediment beds, even when the suspended particles are very small and have no appreciable settling velocity [70]. The rate at which hyporheic exchange occurs is dependent on several conditions, including the type of sediment and the particle surface chemistry. Indeed, EcO157 has been shown to survive for extended periods in sediment, becoming undetectable only after 60 days at 24°C [65]. In a separate study, however, investigation of the Oldman River Basin in Canada for watershed contamination with coliforms indicated that most contamination was coming from a variety of non-point sources and not from sediment [71]. In contrast, another study of the Oak Creek in Arizona indicated that seasonal fecal coliform levels were associated predominantly with sediment agitation during summer storms and recreational activity [72]. Thus, the contribution made by EcO157 sequestered in the sediment to the dynamics of stream contamination appears complicated by a variety of factors, some specific to the region being studied.

Although EcO157 was at increased incidence during and after heavy rain events and flooding, EcO157 was also isolated during periods of drought. For example, EcO157 was isolated from July 2004 samples from Farm A and one year later from Gabilan Creek samples. In both locations, cattle, horses and other livestock had direct access to the streams and were within 1.6 km of the sample site. Similarly, EcO157 was isolated in May and July 2006 in the Gabilan and in October 2006 from surface water at F/R A during the spinach outbreak investigation [9]. The samples were again obtained near obvious animal point sources that could provide direct exposure of surface water to animal feces. However, EcO157 was also recovered August 2006 from the Salinas River (Figure 1, location O), where no obvious source within 8 km was noted. Furthermore, the flow rate in the Salinas River on the sample date was a relatively fast 3100 liter/s (average of gage readings 14 km upstream and 10.5 km downstream). Therefore, the probability of detecting contamination in such a large volume of water is low considering the dilution effect. Detection of the pathogen in large volumes of water will depend upon the sensitivity of the isolation procedure, the number of point sources, and/or the concentrations of viable pathogen in the sources. Nevertheless, these results, in conjunction with MLVA typing data (see below), suggest possible survival and transport of viable pathogen for extended periods and over 16–30 km distances, even during the drought season. However, the potential contribution of multiple point sources of strains indistinguishable from those at distant sites can not be eliminated.

### Incidence of coliforms and generic *E. coli*

Water samples were analyzed also for coliforms and generic *E. coli* to assess the total maximum daily load (TMDL) in the watershed. The results confirmed that the Salinas Valley watershed would be considered “impaired”, since>10% of the samples exceeded coliform levels of 400 MPN/100 ml, and many samples exceeded *E. coli* levels of 404 MPN/100 ml [42]. The incidence of multiple strains of EcO157 at multiple times of the year and at multiple sites in the watershed is consistent with this designation. Our analysis of generic *E. coli* levels from duplicate watershed samples indicated a correlation with the incidence of EcO157. Samples showing high levels of generic *E. coli* were more likely to contain EcO157 (P = 0.001) when all samples were included in the analysis. However, the correlation was insignificant at most individual sample sites due to a very low incidence of EcO157. Beyond the Gabilan drainage EcO157 was undetectable in many samples with high levels of generic *E. coli*. In general, generic *E. coli* is a poor indicator of EcO157.

### MLVA of EcO157 strains and comparison to PFGE

Multiple strains of EcO157, isolated from samples obtained from farms and ranches associated with outbreaks and from the Salinas Valley watershed, were typed by MLVA and PFGE. Our experience confirmed that MLVA was more efficient than PFGE for analysis of a large number of strains obtained during complex investigations. The ability to MLVA type multiple suspect CFU and to distinguish between closely related strains facilitated a rapid assessment of strain relatedness for purposes of source-tracking. For example, during the spinach F/R investigation, the USDA-ARS laboratory identified 27 environmental samples positive for EcO157 and subcultured up to 40 suspect CFU per sample for further analysis. Of greater than 300 isolates confirmed as EcO157 and typed by MLVA, 32 different MLVA types were identified. Many of the 32 MLVA types were closely related based on single TR differences (Table S1), but at least two strains, assessed as different by MLVA, were identified from 8 of the 27 samples (30%). Furthermore, 15 of the 32 MLVA types, identified as closely related to the major MLVA type for spinach outbreak clinical strains (MLVA 163), were all indistinguishable by two enzyme PFGE (PulseNet profile EXHX01.0124-EXHA26.0015). Although genotyping of multiple isolates from each clinical sample is not necessary in most cases, environmental samples provide a greater potential diversity of strains of a target species, due to increased exposure to multiple potential sources of pathogen contamination (e.g. wildlife, compost/manure, water, dust, feed). A similar study to assess multiple suspect isolates by PFGE would be much more labor intensive, costly, and less discriminatory compared to MLVA.

Of the 92 MLVA types identified during this study, 32 types (35% of the total) were isolated exclusively from the Gabilan watershed or its tributaries, and another 32 types were isolated during the 2006 spinach outbreak investigation, including 23 types isolated exclusively from F/R A [43]. Similarly, nine MLVA types were for strains isolated exclusively from F/R B. As noted above, many of the strains isolated from specific locations or sample dates (e.g., intra-farm/ranch or watershed) are related closely (Figure 7, Table S1). For the purpose of this analysis, strains considered closely related have variations in only one or two MLVA loci with small variations in the number of TR. This relationship between MLVA types is based on the assumption that variation in the number of TR occurs most often with changes of one or two repeat units [36]. Often PFGE data supported assumptions of relatedness, in that MLVA clusters were often also represented in the PFGE trees, although, usually, a single PFGE profile was associated with multiple MLVA types. Nevertheless, more research is needed in the molecular biology of TR variation, especially for MLVA typing of strains isolated from environmental samples and/or clinical strains not connected epidemiologically, spatially or temporally.

Temporal and spatial clustering of related MLVA types suggested that, in general, movement of the bacteria in the environment is restricted. For example, identical or closely related MLVA types were not identified as randomly dispersed in the region studied. More than 70% of the strains were clustered spatially (<8 km or further if hydrologically linked). Strains indistinguishable by MLVA type and isolated from separate locations (sometimes>16 km), suggest probable transport of pathogen via linked watersheds. Probable watershed transport of EcO157 was indicated most clearly by investigation of a point source involving cattle in a small corral surrounding a portion of upper Towne Creek (Figures 1 and 6). All of the EcO157 strains isolated within 135 meters downstream from this potential source and strains isolated from the corral (MLVA types143 and 145) were related closely by MLVA (Table S1 and Figure 6), suggesting the corral as the probable source of contamination. Several of the MLVA types were isolated from a only one of the sites, and only from one or two of the four) samples obtained, again reflecting either limited sensitivity of the isolation procedure and/or the dynamic nature of the contamination. Nevertheless, the number of different strains recovered along the stream was proportional inversely to the distance from the point source, and one strain (MLVA type 143) was present at all sample sites up to 135 meters from the corral. Two other strains represented by MLVA types 145 and 147 were isolated only from samples collected up to 64 meters away, possibly because they were at a lower initial concentration compared to MLVA type 143. Nevertheless, potential transport by unknown mechanisms, unrelated to water, with dissemination to multiple sites cannot be eliminated as a possible explanation for the spatial re-occurrence of strains.

MLVA data suggest also long distance transport of the pathogen occurs. Identical types (supported by both MLVA and PFGE data) were found up to 24 km apart and always in the same drainage. Furthermore, isolates suspected of being closely related were recovered often from sites greater than 8 km apart, but nearly always in the same drainage. Other studies on the persistence of pathogens in a water source, modeled using indicator organisms, have examined the effects of a number of attenuation parameters such as temperature, flow rate, particle size, pH, and sediment type [73], [74]. The general conclusions from these studies is that bacteria are transported in watersheds relatively far (km), limited primarily by their ability to survive and remain suspended for movement in a watershed. MLVA types in clusters B and G isolated during the winter of 2006 and 2005, respectively (Figures 5 and 7), are of particular interest, since nearly all strains re-isolated more than 16 km apart are contained in these two clusters. The relatedness of strains isolated from distant sites might reflect an increased fitness for survival in the watershed compared to other strains, consistent with a report of STEC strains varying in length of survival in well water [75]. Alternately, the isolates in clusters B and G may have been at high concentrations initially in or near the Gabilan Creek, thereby increasing the probability of isolating them again downstream even after significant dilution. Increased fitness of these strains in the GI tracts of wild or domestic animals may lead to high concentrations in feces for further dissemination in the environment, increased incidence, and potential increased importance to epidemiology of outbreaks [59].

Strains related by MLVA type might be introduced into an environment from multiple sources and independent of watersheds. However, transport of bacteria by a non-watershed mechanism would be expected to disperse bacteria more randomly in the watershed than was observed. Identical strains of EcO157 were only isolated where a hydrological connection existed. Our data support the conclusion that transport of EcO157 in the Salinas region is due primarily to water. Nevertheless, two clusters of related MLVA types represented strains isolated from locations not linked hydrologically (Figure 7). MLVA cluster B contained isolates from both the Alisal (MLVA types 100, 105) and Gabilan Creeks (MLVA types 93, 101, 102 103), which are connected only where water from both enter into the Reclamation Canal (Figure 1). Additionally, MLVA cluster H strains were isolated from F/R A (MLVA types 189, 196; several sources) and F/R B (MLVA 191, 194; cattle feces). These two F/Rs are approximately 55 km apart and are not connected hydrologically (Figure 4). MLVA types represented by clusters B and H also are clustered based on PFGE data (98 and 88% similarity, respectively). Nevertheless, transport of EcO157 outside of the watershed is probably occurring also. Obvious mechanisms are through wildlife and livestock movement or transport, and contaminated feed [76].

Prior to the spinach outbreak investigation, MLVA comparisons between environmental, and a select group of clinical (sporadic and outbreak) isolates, revealed no identical matches. Traceback data obtained during the spinach investigation narrowed the potential F/R sites to four, and intensive sampling at one of the ranches (F/R A) yielded two isolates identical to the major human outbreak strain by MLVA, but also>30 additional isolates that were related closely by MLVA (Figure 7, Table S1). At least 13 different MLVA types were isolated from feral swine feces and 6 of the MLVA types were closely related to the outbreak MLVA type (MLVA 163) and had PFGE profiles indistinguishable from the outbreak strains (Figure 8, cluster C). These results indicate that roaming feral swine could transport pathogens from multiple point sources to other locations. The prevalence of feral swine near some leafy vegetable production regions, and their wide range and lack of containment, emphasize the need for additional studies to determine their incidence in other regions, potential for colonization by EcO157 and concentration in feces. A more complete analysis of the incidence of EcoliO157 and population density of feral swine related to the 2006 spinach outbreak will be provided in a separate report (Jay MT, Cooley MB, Carychao D, Wiscomb GW, Sweitzer RA, et al. (2007) *Escherichia coli* O157:H7 in feral swine near spinach fields and cattle, central California coast, Emerg. Infect. Dis. (Accepted for publication)).

### Comparison of MVLA types and PFGE profiles to data in PulseNet

Comparisons of the PFGE patterns of our environmental isolates with the PulseNet database revealed several exact matches. It is especially intriguing that three PFGE profiles were associated in PulseNet with multiple multi-state outbreaks occurring in 2003, 2004, 2005 and 2006 (Table 1). Nevertheless, a comparison of MLVA types for eight environmental or clinical isolates with identical PFGE (*Xba*I-*Bln*I) profiles (Table 2), revealed that the strains were less related by MLVA than indicated by PFGE. However, it is recognized also that subtle differences in TR may be determined in different laboratories (Table 2: strains VA-01-577 and F7410) due to differences in reagents, instrumentation and analysis. Additionally, it is important to note that matching PFGE profiles for strains not linked epidemiologically (i.e. environmental strains from this study and PulseNet-related strains), should be interpreted with caution. This is especially true since digestion patterns from six or more enzymes are considered necessary for an accurate estimation of relatedness with PFGE [76]. However, MLVA should provide a more accurate assessment of strain-relatedness between non-epidemiologically-related strains than PFGE. A robust MLVA database would assist in making links between environmental and clinical isolates. Nevertheless, higher resolution genotyping methods, such as single nucleotide polymorphism (SNP) arrays [77], [78] or partial genome sequencing of strains, would be most beneficial for accurate source tracking and improved epidemiology during investigation of outbreaks and sporadic illness.

The frequent recovery of EcO157 from a variety of distant sites in the Salinas watershed and the spinach outbreak F/Rs, and also the isolation of identical, or closely related MLVA types from the same locations, days or months apart, indicate that contamination may be more prevalent than assumed previously. The most likely explanation for these results is re-introduction from one or more sources, but long-term survival of the pathogen in the environment is also possible, stimulating important questions regarding the source and mechanisms of survival, growth, and prevalence of EcO157 in these environments [14], [79]–[83]. Understanding the factors related to EcO157 survival and growth in the leafy vegetable production environments, along with fate and transport processes, is critical to developing intervention strategies that can minimize the probability of an outbreak associated with bagged leafy vegetables.

## Supporting Information

Table S1 Strains used in this study. (0.41 MB DOC)
