# Supplementary material for: Incidence and Tracking of Escherichia coli O157:H7 in a Major Produce Production Region in California
Source: PLoS One. 2007 Nov 14;2(11):e1159. doi: 10.1371/journal.pone.0001159 (PMC2174234; doi:10.1371/journal.pone.0001159)
Supplement: Table S1 — (0.41 MB DOC) [file pone.0001159.s001.doc]

**Table S1. Strains used in this study.**

| **Location^a^** | **Date** | **Source^b^** | **“RM” strain#** | **MLVA# ^c^** | **Stx1^d^** | **Stx2^d^** | **Genotype^e^** |
| --- | --- | --- | --- | --- | --- | --- | --- |
| Farm A | 07/13/2004 | Water-Sediment | 4403 | 17 | - | + | 28-10-X-17-7-9-5-3-6-6 |
| A* | 04/18/2006 | Swab | 5681 | 16 | + | + | 30-10-11-15-7-9-9-9-4-7 |
| A* | 04/18/2006 | Water | 5714 | 16 | + | + | 30-10-11-15-7-9-9-9-4-7 |
| A* | 11/30/2005 | Water | 5450 | 89 | + | + | 31-10-11-15-7-9-9-9-4-7 |
| A* | 03/15/2006 | Water | 5621 | 89 | + | + | 31-10-11-15-7-9-9-9-4-7 |
| A* | 04/05/2006 | Water | 5665 | 89 | + | + | 31-10-11-15-7-9-9-9-4-7 |
| A* | 04/18/2006 | Water | 5685 | 89 | + | + | 31-10-11-15-7-9-9-9-4-7 |
| A* | 02/22/2006 | Swab | 5615 | 97 | - | + | 23-6-X-14-4-9-6-3-7-7 |
| A* | 03/20/2006 | Swab | 5626 | 97 | - | + | 23-6-X-14-4-9-6-3-7-7 |
| A* | 02/22/2006 | Swab | 5616 | 98 | - | + | 24-6-X-14-4-9-6-3-7-7 |
| A* | 03/07/2006 | Water | 5620 | 99 | + | + | 30-10-11-14-7-9-9-9-4-7 |
| A* | 04/18/2006 | Swab | 5676 | 106 | + | + | 25-10-12-15-7-9-9-9-4-7 |
| A* | 04/18/2006 | Swab | 5677 | 107 | + | + | 29-10-11-15-7-9-9-9-4-7 |
| A* | 04/18/2006 | Swab | 5678 | 108 | + | + | 31-10-12-15-7-9-9-9-4-7 |
| A* | 04/18/2006 | Swab | 5718 | 122 | + | + | 20-10-11-15-7-9-9-9-4-7 |
| A* | 05/15/2006 | Water | 5725 | 143 | + | + | 30-10-12-14-7-9-9-9-4-7 |
| A* | 05/23/2006 | Water | 5734 | 143 | + | + | 30-10-12-14-7-9-9-9-4-7 |
| A* | 05/31/2006 | Sediment | 5853 | 143 | + | + | 30-10-12-14-7-9-9-9-4-7 |
| A* | 05/15/2006 | Water | 5726 | 144 | + | + | 31-10-12-14-7-9-9-9-4-7 |
| A* | 05/23/2006 | Water | 5751 | 145 | + | + | 32-10-12-14-7-9-9-9-4-7 |
| A* | 05/31/2006 | Sediment | 5855 | 145 | + | + | 32-10-12-14-7-9-9-9-4-7 |
| A* | 05/15/2006 | Water | 5728 | 146 | + | + | 33-10-12-14-7-9-9-9-4-7 |
| A* | 05/23/2006 | Water | 5748 | 146 | + | + | 33-10-12-14-7-9-9-9-4-7 |
| A* | 05/23/2006 | Water | 5739 | 147 | + | + | 21-10-12-14-7-9-9-9-4-7 |
| A* | 05/23/2006 | Water | 5756 | 148 | + | + | 24-10-12-14-7-9-9-9-4-7 |
| A* | 05/23/2006 | Water | 5740 | 149 | + | + | 29-10-12-14-7-9-9-9-4-7 |
| A* | 05/23/2006 | Water | 5736 | 150 | + | + | 30-10-12-15-7-9-9-9-4-7 |
| A* | 05/23/2006 | Water | 5754 | 151 | + | + | 30-10-13-14-7-9-9-9-4-7 |
| A* | 05/23/2006 | Water | 5752 | 152 | + | + | 31-10-13-14-7-9-9-9-4-7 |
| A* | 08/22/2006 | Swab | 6009 | 159 | + | + | 32-10-12-14-7-9-9-9-4-8 |
| A* | 08/22/2006 | Swab | 6005 | 162 | + | + | 30-10-12-14-7-9-9-9-4-8 |
| B* | 08/16/2005 | Cow-Fecal | 5038 | 15 | + | + | 30-10-11-16-7-9-9-9-4-7 |
| B* | 08/16/2005 | Cow-Fecal | 5037 | 16 | + | + | 30-10-11-15-7-9-9-9-4-7 |
| B* | 04/05/2006 | Water | 5670 | 93 | - | + | 20-9-11-14-7-9-8-4-7-6 |
| B* | 04/05/2006 | Water | 5673 | 102 | - | + | 21-9-11-14-7-9-8-4-7-6 |
| B* | 05/15/2006 | Swab | 5850 | 154 | + | + | 29-10-10-10-5-9-4-4-11-3 |
| B* | 07/18/2006 | Swab | 5875 | 155 | + | + | 30-10-10-10-5-9-4-4-10-3 |
| B* | 07/18/2006 | Swab | 5876 | 156 | + | + | 16-10-10-14-7-9-9-9-4-8 |
| B* | 07/18/2006 | Swab | 5877 | 157 | + | + | 29-10-11-10-5-9-4-4-10-3 |
| C* | 07/26/2005 | Water | 5036 | 15 | + | + | 30-10-11-16-7-9-9-9-4-7 |
| C* | 04/05/2006 | Water | 5661 | 89 | + | + | 31-10-11-15-7-9-9-9-4-7 |
| C* | 04/18/2006 | Swab | 5682 | 89 | + | + | 31-10-11-15-7-9-9-9-4-7 |
| C* | 03/20/2006 | Swab | 5624 | 93 | - | + | 20-9-11-14-7-9-8-4-7-6 |
| C* | 04/05/2006 | Water | 5667 | 93 | - | + | 20-9-11-14-7-9-8-4-7-6 |
| C* | 03/20/2006 | Swab | 5625 | 102 | - | + | 21-9-11-14-7-9-8-4-7-6 |
| C* | 04/18/2006 | Swab | 5679 | 109 | + | + | 32-10-11-15-7-9-9-9-4-7 |
| C* | 05/15/2006 | Water | 5721 | 142 | + | + | 29-10-10-10-5-9-4-4-10-3 |
| C* | 07/18/2006 | Water | 5872 | 142 | + | + | 29-10-10-10-5-9-4-4-10-3 |
| C* | 08/22/2006 | Swab | 6008 | 158 | + | + | 31-10-12-14-7-9-9-9-4-8 |
| D* | 02/16/2005 | Water | 4863 | 6 | + | + | 15-11-11-10-7-9-10-10-5-9 |
| D* | 03/23/2005 | Water | 4884 | 9 | + | + | 11-9-10-12-5-9-4-4-10-3 |
| D* | 03/23/2005 | Water | 4886 | 11 | + | + | 11-10-10-12-5-6-2-5-5-4 |
| D* | 04/05/2006 | Water | 5662 | 89 | + | + | 31-10-11-15-7-9-9-9-4-7 |
| D* | 04/18/2006 | Swab | 5683 | 89 | + | + | 31-10-11-15-7-9-9-9-4-7 |
| D* | 03/20/2006 | Water | 5627 | 99 | + | + | 30-10-11-14-7-9-9-9-4-7 |
| E* | 02/16/2005 | Water | 4869 | 2 | + | + | 14-11-11-10-7-9-10-10-5-9 |
| E* | 04/05/2006 | Water | 5660 | 16 | + | + | 30-10-11-15-7-9-9-9-4-7 |
| E* | 04/05/2006 | Water | 5663 | 89 | + | + | 31-10-11-15-7-9-9-9-4-7 |
| E* | 04/18/2006 | Swab | 5684 | 89 | + | + | 31-10-11-15-7-9-9-9-4-7 |
| E* | 03/20/2006 | Swab | 5622 | 93 | - | + | 20-9-11-14-7-9-8-4-7-6 |
| E* | 03/20/2006 | Swab | 5630 | 101 | - | + | 20-9-11-13-7-9-8-4-7-6 |
| E* | 03/20/2006 | Swab | 5623 | 103 | - | + | 20-10-11-14-7-9-8-4-7-6 |
| F* | 02/16/2005 | Water | 4875 | 4 | + | + | 23-11-12-11-7-9-13-7-4-5 |
| F* | 03/23/2005 | Water | 4878 | 7 | - | - | 13-10-14-12-7-6-2-5-5-4 |
| G* | 02/16/2005 | Water | 4859 | 1 | + | + | 14-11-11-12-7-9-10-10-5-9 |
| G* | 02/16/2005 | Water | 4861 | 2 | + | + | 14-11-11-10-7-9-10-10-5-9 |
| G* | 03/23/2005 | Water | 4882 | 8 | + | + | 22-10-10-10-5-9-4-4-10-3 |
| G* | 03/23/2005 | Water | 4888 | 13 | + | + | 13-11-11-12-7-9-10-10-5-9 |
| G* | 03/23/2005 | Water | 4889 | 14 | + | + | 24-10-10-10-5-9-4-4-10-3 |
| G* | 04/05/2006 | Water | 5672 | 93 | - | + | 20-9-11-14-7-9-8-4-7-6 |
| G* | 04/18/2006 | Swab | 5687 | 97 | - | + | 23-6-X-14-4-9-6-3-7-7 |
| G* | 03/20/2006 | Swab | 5628 | 100 | - | + | 18-9-11-13-7-9-8-4-7-6 |
| H* | 02/16/2005 | Water | 4862 | 3 | + | + | 17-11-11-12-7-9-10-10-5-9 |
| H* | 02/16/2005 | Water | 4860 | 4 | + | + | 23-11-12-11-7-9-13-7-4-5 |
| H* | 03/23/2005 | Water | 4885 | 10 | + | + | 13-10-14-12-7-9-4-4-10-3 |
| H* | 03/23/2005 | Water | 4887 | 12 | + | + | 11-10-10-12-5-9-4-4-10-3 |
| H* | 04/18/2006 | Water | 5691 | 100 | - | + | 18-9-11-13-7-9-8-4-7-6 |
| I* | 02/16/2005 | Water | 4866 | 2 | + | + | 14-11-11-10-7-9-10-10-5-9 |
| I* | 02/16/2005 | Water | 4864 | 5 | + | + | 14-11-12-10-7-9-10-10-5-9 |
| I* | 03/23/2005 | Water | 4883 | 9 | + | + | 11-9-10-12-5-9-4-4-10-3 |
| J* | 01/04/2006 | Water | 5603 | 90 | - | + | 25-9-7-13-7-6-5-4-6-4 |
| J* | 01/04/2006 | Water | 5604 | 91 | - | + | 5-9-X-9-4-9-X-3-6-7 |
| J* | 01/04/2006 | Water | 5605 | 92 | - | + | 27-11-15-14-7-9-7-7-4-7 |
| J* | 01/17/2006 | Swab | 5606 | 93 | - | + | 20-9-11-14-7-9-8-4-7-6 |
| J* | 01/17/2006 | Swab | 5607 | 94 | + | + | 34-11-18-17-7-9-11-8-6-7 |
| J* | 01/17/2006 | Swab | 5608 | 95 | + | + | 35-11-18-17-7-9-11-8-6-7 |
| J* | 01/17/2006 | Swab | 5609 | 96 | + | + | 34-11-19-17-7-9-11-8-6-7 |
| K* | 04/18/2006 | Swab | 5689 | 100 | - | + | 18-9-11-13-7-9-8-4-7-6 |
| L* | 04/18/2006 | Swab | 5690 | 100 | - | + | 18-9-11-13-7-9-8-4-7-6 |
| M* | 04/05/2006 | Water | 5674 | 104 | + | + | 24-10-10-12-5-9-5-4-11-3 |
| N* | 04/18/2006 | Water | 5688 | 100 | - | + | 18-9-11-13-7-9-8-4-7-6 |
| N* | 04/18/2006 | Water | 5675 | 105 | - | + | 18-9-11-14-7-9-8-4-7-6 |
| O* | 08/22/2006 | Water | 6002 | 160 | - | + | 25-9-8-10-X-9-13-5-7-10 |
| O* | 08/22/2006 | Water | 6003 | 161 | - | + | 26-9-8-10-X-9-13-5-7-10 |
| CAN | 1988 | Human | 2070 | 41 | - | + | 22-10-17-12-2-9-6-3-6-6 |
| DEN | 1988 | Human | 2071 | 26 | + | - | 13-9-17-14-7-9-4-8-6-7 |
| DEN | 1987 | Human | 2026 | 28 | - | + | 14-9-12-11-7-9-4-4-9-8 |
| CA | 1997 | Human-S | 1241 | 27 | - | + | 14-9-8-10-X-8-13-5-7-9 |
| CA | 1997 | Human-S | 1243 | 35 | + | + | 18-11-13-18-7-9-10-9-6-7 |
| CA | 1997 | Human-S | 1240 | 44 | + | + | 27-11-11-12-7-9-10-9-5-10 |
| CA | 1997 | Human-S | 1242 | 54 | + | + | 35-12-10-13-7-9-6-7-6-7 |
| CA | 2005 | Human-S | 5202 | 55 | - | + | 12-9-14-8-7-9-8-4-7-7 |
| CA | 2005 | Human-S | 5184 | 56 | + | + | 12-11-10-18-7-9-8-7-4-11 |
| CA | 2005 | Human-S | 5188 | 57 | + | + | 12-11-10-20-7-9-8-7-4-11 |
| CA | 2005 | Human-S | 5207 | 58 | - | + | 15-9-11-11-7-9-8-4-6-5 |
| CA | 2005 | Human-S | 5192 | 59 | + | + | 15-11-10-21-7-9-8-8-4-10 |
| CA | 2005 | Human-S | 5200 | 60 | - | + | 17-9-11-15-7-9-8-4-7-6 |
| CA | 2005 | Human-S | 5196 | 62 | + | + | 19-11-11-14-7-9-10-8-4-7 |
| CA | 2005 | Human-S | 5185 | 63 | - | + | 20-8-14-16-7-9-8-4-7-8 |
| CA | 2005 | Human-S | 5187 | 64 | - | + | 20-9-16-20-7-9-4-4-7-6 |
| CA | 2005 | Human-S | 5206 | 65 | + | + | 21-11-11-17-7-9-11-8-4-6 |
| CA | 2005 | Human-S | 5189 | 66 | + | + | 22-11-10-9-7-9-11-9-4-7 |
| CA | 2005 | Human-S | 5186 | 67 | + | + | 24-11-17-20-7-9-11-7-4-6 |
| CA | 2005 | Human-S | 5193 | 68 | + | + | 25-11-13-10-7-9-9-7-4-5 |
| CA | 2005 | Human-S | 5197 | 69 | + | + | 25-11-13-10-7-9-10-7-4-5 |
| CA | 2005 | Human-S | 5204 | 70 | + | + | 27-11-12-12-7-9-11-8-6-8 |
| CA | 2005 | Human-S | 5190 | 71 | - | + | 30-12-14-10-7-9-7-8-4-5 |
| CA | 2005 | Human-S | 5194 | 72 | - | + | 34-9-18-15-7-9-9-4-7-6 |
| CA | 2005 | Human-S | 5205 | 73 | + | + | 35-12-10-7-7-9-7-7-6-8 |
| CA | 2005 | Human-S | 5198 | 74 | + | + | 44-12-10-15-7-9-6-7-7-7 |
| CA | 07/03/2005 | Human-S | 5330 | 76 | + | + | 24-11-8-12-7-9-9-7-4-6 |
| CA | 09/18/2005 | Human-S | 5331 | 77 | + | + | 13-11-14-18-7-9-11-7-4-6 |
| CA | 09/04/2005 | Human-S | 5332 | 78 | + | + | 20-11-14-19-7-9-10-9-6-7 |
| CA | 09/04/2005 | Human-S | 5333 | 79 | - | + | 26-11-11-12-7-9-10-7-4-5 |
| CA | 01/16/2005 | Human-S | 5337 | 80 | - | + | 29-9-18-17-7-9-5-4-8-6 |
| CA | 08/01/2005 | Human-S | 5338 | 81 | + | + | 18-12-10-15-7-9-6-7-6-6 |
| CA | 09/23/2005 | Human-S | 5396 | 82 | + | + | 17-11-10-12-7-9-13-7-4-7 |
| CA | 08/27/2004 | Human-S | 5405 | 83 | + | + | 18-11-16-18-7-9-11-8-5-8 |
| CA | 08/21/2004 | Human-S | 5399 | 84 | + | + | 22-11-10-13-7-9-9-7-4-7 |
| CA | 08/13/2004 | Human-S | 5398 | 85 | + | + | 25-11-14-19-7-9-9-7-6-8 |
| CA | 08/30/2004 | Human-S | 5402 | 86 | + | + | 27-10-11-15-7-9-9-9-4-7 |
| CA | 08/17/2004 | Human-S | 5404 | 87 | - | + | 32-11-11-11-7-9-10-8-4-7 |
| CA | 08/22/2004 | Human-S | 5403 | 88 | + | + | 33-11-12-16-7-9-9-12-7-7 |
| CA | 04/27/2006 | Human-S | 5642 | 110 | + | + | 9-11-11-18-7-9-11-6-4-8 |
| CA | 2006 | Human-S | 5645 | 111 | - | + | 10-10-15-14-7-6-6-5-5-6 |
| CA | 2006 | Human-S | 5657 | 112 | - | + | 12-9-7-13-7-6-5-4-6-4 |
| CA | 04/27/2006 | Human-S | 5650 | 113 | + | + | 13-11-14-19-7-9-12-7-4-6 |
| CA | 2006 | Human-S | 5643 | 114 | - | + | 15-9-11-16-7-9-6-4-8-6 |
| CA | 2006 | Human-S | 5652 | 115 | - | + | 15-9-11-18-7-9-9-4-7-6 |
| CA | 2006 | Human-S | 5658 | 116 | - | + | 16-9-11-13-7-9-9-4-7-6 |
| CA | 04/27/2006 | Human-S | 5706 | 117 | + | + | 18-12-11-6-7-9-8-7-7-6 |
| CA | 10/19/2005 | Human-S | 5699 | 118 | + | + | 19-11-10-12-7-9-12-6-4-11 |
| CA | 2006 | Human-S | 5637 | 119 | + | + | 20-11-8-13-7-9-6-11-6-7 |
| CA | 2006 | Human-S | 5646 | 120 | - | + | 20-9-9-12-2-8-X-5-7-11 |
| CA | 10/25/2005 | Human-S | 5700 | 121 | + | + | 20-11-10-12-7-9-12-6-4-11 |
| CA | 2006 | Human-S | 5656 | 123 | - | + | 20-9-13-10-7-6-6-5-4-6 |
| CA | 2006 | Human-S | 5647 | 124 | - | + | 21-9-9-10-2-8-6-4-7-10 |
| CA | 2006 | Human-S | 5639 | 125 | + | + | 22-X-12-8-7-9-8-7-6-7 |
| CA | 02/06/2007 | Human-S | 5702 | 126 | + | + | 23-11-11-15-7-9-X-9-6-7 |
| CA | 2006 | Human-S | 5655 | 127 | - | + | 23-11-13-15-7-9-10-7-6-6 |
| CA | 2006 | Human-S | 5659 | 128 | + | + | 24-11-13-18-7-9-14-7-4-6 |
| CA | 2006 | Human-S | 5640 | 129 | + | + | 25-11-12-11-7-9-12-7-4-5 |
| CA | 2006 | Human-S | 5636 | 130 | + | + | 26-11-10-16-7-9-11-7-6-9 |
| CA | 2006 | Human-S | 5653 | 131 | + | + | 26-11-13-12-7-9-9-7-4-6 |
| CA | 10/05/2005 | Human-S | 5697 | 132 | + | + | 27-11-12-15-7-9-X-7-6-7 |
| CA | 2006 | Human-S | 5648 | 133 | + | + | 28-11-12-12-7-9-11-7-6-7 |
| CA | 2006 | Human-S | 5649 | 134 | + | + | 30-10-14-15-7-6-7-5-4-7 |
| CA | 2006 | Human-S | 5654 | 135 | - | + | 32-9-23-11-2-9-X-6-8-10 |
| CA | 2006 | Human-S | 5644 | 136 | - | + | 34-9-11-6-7-9-9-4-7-6 |
| CA | 09/22/2005 | Human-S | 5696 | 137 | - | + | 35-9-16-16-7-9-12-4-7-6 |
| CA | 2006 | Human-S | 5635 | 138 | + | + | 37-10-14-14-7-9-10-7-7-5 |
| CA | 2006 | Human-S | 5638 | 139 | - | + | 39-9-17-10-2-9-X-5-8-8 |
| CA | 2006 | Human-S | 5641 | 140 | + | + | 41-12-11-13-8-9-6-7-6-8 |
| CA | 2006 | Human-S | 5651 | 141 | - | + | X-9-16-X-2-9-X-5-8-8 |
| NM | 1988 | Human-S | 3649 | 51 | + | + | 18-12-11-15-7-9-10-7-6-8 |
| PA | 2006 | Human-S | 6056 | 165 | - | + | 10-9-22-14-7-9-6-4-9-5 |
| PA | 2006 | Human-S | 6051 | 167 | - | + | 20-9-13-15-7-9-8-4-7-7 |
| PA | 2006 | Human-S | 6059 | 168 | - | + | 31-9-6-15-7-9-4-4-8-11 |
| PA | 2006 | Human-S | 6054 | 169 | - | + | 33-9-14-19-7-9-8-4-7-6 |
| PA | 2006 | Human-S | 6055 | 170 | - | + | 37-9-16-12-7-9-8-4-7-8 |
| PA | 2006 | Human-S | 6053 | 171 | - | + | 38-9-14-18-7-9-10-4-7-7 |
| PA | 2006 | Human-S | 6066 | 172 | - | + | 54-9-18-16-7-9-8-4-7-6 |
| CAN | 1991 | Human-S | 3650 | 50 | + | - | 18-12-10-15-7-9-10-7-6-8 |
| CAN | 1992 | Human-S | 3651 | 52 | + | - | 18-12-14-14-7-9-10-7-6-8 |
| CAN | 1994 | Human-S | 3652 | 38 | + | + | 20-11-11-17-7-9-11-7-6-7 |
| CAN | 1994 | Human-S | 3644 | 40 | - | + | 22-9-4-6-7-9-5-3-6-7 |
| CAN | 1999 | Human-S | 3643 | 49 | + | + | X-11-12-15-7-9-8-X-8-6 |
| CA | 1996 | Food | 1484 | 23 | + | + | 22-11-11-12-7-9-9-8-4-7 |
| MI | 1988 | Human | 2055 | 47 | + | + | 32-11-14-20-7-9-7-6-6-8 |
| NM | 1988 | Human | 2040 | 39 | + | + | 20-11-12-22-7-9-7-7-6-9 |
| Unknown | Unknown | Human | 1273 | 53 | - | - | 30-11-13-13-7-9-6-6-8-6 |
| WA | 1990 | Human | 2025 | 43 | + | + | 27-11-10-21-7-9-10-6-6-8 |
| WA | 1985 | Human | 2010 | 48 | + | + | 42-11-12-19-7-9-9-6-6-8 |
| WA | 07/17/2002 | Human | 4689 | 153 | + | + | X-9-22-X-X-9-13-7-6-7 |
| CAN | 1991 | Human-HUS | 3640 | 30 | + | + | 16-11-12-18-7-9-4-6-6-7 |
| CAN | 1992 | Human-HUS | 3656 | 45 | - | + | 29-11-11-19-7-9-10-6-6-7 |
| CAN | 1993 | Human-HUS | 3641 | 37 | + | - | 18-12-11-16-7-9-10-7-6-6 |
| CAN | 1996 | Human-HUS | 3639 | 46 | - | + | 31-9-19-13-X-9-13-4-8-7 |
| OR | 1983 | Human-OB1 | 2084 | 36 | + | + | 18-12-10-12-7-9-8-X-6-7 |
| CA | 1996 | Human-OB2 | 1239 | 42 | + | - | 23-11-11-12-7-9-9-8-4-7 |
| NE | 02/01/1999 | Human-OB3 | 1918 | 31 | - | + | 17-9-14-12-X-9-13-5-9-8 |
| NE | 02/01/1999 | Human-OB3 | 1930 | 32 | - | + | 17-9-15-12-X-9-13-5-9-8 |
| NE | 02/01/1999 | Human-OB3 | 1915 | 33 | - | + | 17-11-15-12-X-9-13-5-9-8 |
| NE | 02/01/1999 | Human-OB3 | 1924 | 34 | - | + | 18-9-15-12-X-9-13-5-9-8 |
| CA | 1999 | Human-OB4 | 2189 | 25 | + | + | 12-11-11-12-7-9-11-8-5-7 |
| CA | 1999 | Human-OB4 | 2193 | 29 | + | + | 16-11-11-12-7-9-11-8-5-7 |
| CAN | 2000 | Human-OB5 | 4263 | 24 | + | + | 9-10-12-16-7-9-10-7-5-7 |
| WA | 07/30/2002 | Human-OB6 | 4688 | 21 | + | + | X-9-22-X-X-9-13-5-8-8 |
| WA | 07/30/2002 | Human-OB6 | 4690 | 21 | + | + | X-9-22-X-X-9-13-5-8-8 |
| WA | 07/30/2002 | Human-OB6 | 4691 | 21 | + | + | X-9-22-X-X-9-13-5-8-8 |
| WA | 07/30/2002 | Human-OB6 | 4692 | 21 | + | + | X-9-22-X-X-9-13-5-8-8 |
| CA | 10/03/2003 | Human-OB7 | 4406 | 18 | + | + | 21-11-14-11-7-9-10-8-4-7 |
| CA | 10/03/2003 | Human-OB7 | 5011 | 19 | + | + | 20-11-14-11-7-9-10-8-4-7 |
| CA | 10/01/2003 | Human-OB8 | 4407 | 20 | + | + | 12-11-11-11-7-9-11-X-4-5 |
| MN | 2005 | Human-OB9 | 5277 | 61 | + | + | 17-12-10-11-7-9-12-8-4-7 |
| MN | 2005 | Human-OB9 | 5278 | 61 | + | + | 17-12-10-11-7-9-12-8-4-7 |
| MN | 2005 | Human-OB9 | 5279 | 61 | + | + | 17-12-10-11-7-9-12-8-4-7 |
| MN | 2005 | Human-OB9 | 5280 | 61 | + | + | 17-12-10-11-7-9-12-8-4-7 |
| MN | 2005 | Human-OB9 | 5281 | 61 | + | + | 17-12-10-11-7-9-12-8-4-7 |
| MN | 2005 | Human-OB9 | 5282 | 61 | + | + | 17-12-10-11-7-9-12-8-4-7 |
| **CA** | **2006** | **Human-OB10** | **6049** | **163** | **-** | **+** | **16-9-11-17-7-9-9-4-7-6** |
| **ID** | **2006** | **Human-OB10** | **6045** | **163** | **-** | **+** | **16-9-11-17-7-9-9-4-7-6** |
| **PA** | **2006** | **Human-OB10** | **6057** | **163** | **-** | **+** | **16-9-11-17-7-9-9-4-7-6** |
| **WI** | **2006** | **Human-OB10** | **6011** | **163** | **-** | **+** | **16-9-11-17-7-9-9-4-7-6** |
| **PA** | **2006** | **Spinach-OB10** | **6067** | **163** | **-** | **+** | **16-9-11-17-7-9-9-4-7-6** |
| **ID** | **2006** | **Human-OB10** | **6046** | **164** | **-** | **+** | **17-9-11-17-7-9-9-4-7-6** |
| **PA** | **2006** | **Human-OB10** | **6024** | **164** | **-** | **+** | **17-9-11-17-7-9-9-4-7-6** |
| **PA** | **2006** | **Human-OB10** | **6064** | **166** | **-** | **+** | **16-9-11-15-7-9-8-4-7-8** |
| **F/R A** | **10/25/2006** | **Pig Feces** | **6146** | **116** | **-** | **+** | **16-9-11-13-7-9-9-4-7-6** |
| **F/R A** | **10/03/2006** | **Cow Feces** | **6103** | **163** | **-** | **+** | **16-9-11-17-7-9-9-4-7-6** |
| **F/R A** | **11/13/2006** | **Pig Feces** | **6155** | **163** | **-** | **+** | **16-9-11-17-7-9-9-4-7-6** |
| **F/R A** | **10/25/2006** | **Pig Feces** | **6145** | **173** | **-** | **+** | **14-9-11-13-7-9-9-4-7-6** |
| **F/R A** | **10/03/2006** | **Sediment** | **6105** | **173** | **-** | **+** | **14-9-11-13-7-9-9-4-7-6** |
| **F/R A** | **10/03/2006** | **Water** | **6104** | **173** | **-** | **+** | **14-9-11-13-7-9-9-4-7-6** |
| **F/R A** | **10/03/2006** | **Pig Feces** | **6106** | **174** | **-** | **+** | **14-9-11-16-7-9-9-4-7-5** |
| **F/R A** | **10/03/2006** | **Pig Feces** | **6100** | **175** | **-** | **+** | **14-9-11-16-7-9-9-4-7-6** |
| **F/R A** | **10/03/2006** | **Cow Feces** | **6107** | **176** | **-** | **+** | **14-9-11-17-7-9-9-4-7-6** |
| **F/R A** | **10/03/2006** | **Pig Feces** | **6101** | **176** | **-** | **+** | **14-9-11-17-7-9-9-4-7-6** |
| **F/R A** | **10/24/2006** | **Pig Feces** | **6144** | **176** | **-** | **+** | **14-9-11-17-7-9-9-4-7-6** |
| **F/R A** | **11/13/2006** | **Pig Feces** | **6157** | **176** | **-** | **+** | **14-9-11-17-7-9-9-4-7-6** |
| **F/R A** | **10/03/2006** | **Sediment** | **6110** | **176** | **-** | **+** | **14-9-11-17-7-9-9-4-7-6** |
| **F/R A** | **10/31/2006** | **Soil** | **6149** | **176** | **-** | **+** | **14-9-11-17-7-9-9-4-7-6** |
| **F/R A** | **10/03/2006** | **Water** | **6102** | **176** | **-** | **+** | **14-9-11-17-7-9-9-4-7-6** |
| **F/R A** | **10/03/2006** | **Cow Feces** | **6111** | **177** | **-** | **+** | **14-9-11-17-7-9-9-4-8-6** |
| **F/R A** | **10/03/2006** | **Sediment** | **6112** | **177** | **-** | **+** | **14-9-11-17-7-9-9-4-8-6** |
| **F/R A** | **10/03/2006** | **Sediment** | **6113** | **178** | **-** | **+** | **14-9-11-18-7-9-9-4-8-7** |
| **F/R A** | **2006** | **Cow Feces** | **6092** | **179** | **-** | **+** | **14-9-11-18-7-9-9-4-7-6** |
| **F/R A** | **10/03/2006** | **Pig Feces** | **6099** | **180** | **-** | **+** | **14-9-11-21-7-9-9-4-7-6** |
| **F/R A** | **10/03/2006** | **Cow Feces** | **6114** | **181** | **-** | **+** | **14-9-12-17-7-9-9-4-7-6** |
| **F/R A** | **10/03/2006** | **Sediment** | **6115** | **182** | **-** | **+** | **15-9-11-17-7-9-9-4-8-6** |
| **F/R A** | **2006** | **Cow Feces** | **6095** | **183** | **-** | **+** | **16-9-11-17-7-9-9-4-9-6** |
| F/R A | 10/03/2006 | Pig Feces | 6124 | 189 | - | + | 29-8-X-17-4-9-6-2-6-7 |
| F/R A | 10/03/2006 | Pig Feces | 6097 | 190 | - | + | 29-8-X-17-4-9-6-2-7-7 |
| F/R A | 10/03/2006 | Cow Feces | 6126 | 192 | - | + | 30-8-X-16-4-9-6-2-5-7 |
| F/R A | 10/03/2006 | Cow Feces | 6127 | 193 | - | + | 30-8-X-16-4-9-6-2-4-7 |
| F/R A | 10/03/2006 | Water | 6098 | 195 | - | + | 30-8-X-19-4-9-6-4-6-6 |
| F/R A | 10/03/2006 | Pig Colon | 6129 | 196 | - | + | 31-8-X-18-4-9-6-2-6-7 |
| F/R A | 10/03/2006 | Sediment | 6130 | 196 | - | + | 31-8-X-18-4-9-6-2-6-7 |
| F/R A | 10/03/2006 | Water | 6133 | 197 | - | + | 31-8-X-19-4-9-6-2-6-7 |
| F/R A | 10/03/2006 | Sediment | 6134 | 197 | - | + | 31-8-X-19-4-9-6-2-6-7 |
| **F/R A** | **10/25/2006** | **Pig Feces** | **6148** | **200** | **-** | **+** | **15-9-11-13-7-9-9-4-7-6** |
| **F/R A** | **10/19/2006** | **Pig Feces** | **6143** | **201** | **-** | **+** | **15-9-11-17-7-9-9-4-7-6** |
| F/R A | 11/13/2006 | Pig Feces | 6154 | 202 | + | + | 9-9-10-13-5-9-4-4-10-3 |
| F/R A | 11/13/2006 | Pig Feces | 6158 | 203 | - | + | 33-8-X-18-4-9-6-2-6-7 |
| F/R B | 10/04/2006 | Cow Feces | 6117 | 184 | - | + | 19-9-X-13-4-9-5-2-6-9 |
| F/R B | 10/04/2006 | Cow Feces | 6118 | 185 | + | + | 28-11-15-16-7-9-10-9-6-7 |
| F/R B | 10/04/2006 | Cow Feces | 6119 | 186 | + | + | 28-11-15-17-7-9-10-10-6-7 |
| F/R B | 10/04/2006 | Cow Feces | 6122 | 187 | + | + | 28-11-15-17-7-9-10-9-6-7 |
| F/R B | 10/04/2006 | Cow Feces | 6123 | 188 | + | + | 28-11-16-17-7-9-10-9-6-7 |
| F/R B | 10/04/2006 | Cow Feces | 6125 | 191 | - | + | 29-8-X-18-4-9-6-2-6-7 |
| F/R B | 10/04/2006 | Cow Feces | 6128 | 194 | - | + | 30-8-X-18-4-9-6-2-6-7 |
| F/R B | 10/04/2006 | Cow Feces | 6135 | 198 | - | + | 33-10-X-21-7-9-5-3-6-6 |
| F/R B | 10/04/2006 | Cow Feces | 6136 | 199 | + | + | 35-11-15-17-7-9-10-9-6-7 |

^a^ Single letter codes with an “*” refer to locations designated in Figure 1. Two letter codes designate states in the US where strains were isolated. CAN, Canada; DEN, Denmark; F/R, farm/ranch investigated during 2006 spinach outbreak investigation.

^b^ Human, clinical fecal sample; OB#, outbreak number with # in sequential order of occurrence; S, sporadic illness; swab, Moore swab. OB1, meat (genome strain EDL-933); OB2, apple juice; OB3, iceberg lettuce; OB4, cooked taco meat; OB5, municipal water (“Walkerton Outbreak”); OB6, romaine lettuce, Spokane, WA; OB7, romaine lettuce, San Diego County, CA; OB8, spinach, San Mateo County, CA; OB9, mixed vegetables, bagged; OB10, bagged spinach, multi-state.

^c^ MLVA numbers were assigned in the order they were identified. Strains isolated from samples collected on the same day, and having an identical MLVA type, were omitted from this table.

^d^ Stx1, shigaliketoxin 1; Stx2, shigaliketoxin 2. Indicates presence (+) or absence (-) of the gene sequence identified by PCR.

^e^ MLVA type: “X” indicates the absence of an amplification product for this locus. TR numbers are listed in the following order of loci: Vhec1, Vhec2, Vhec3, Vhec4, Vhec5, Vhec6, Vhec7, O157-17, O157-19, O157-37 (see references noted in Methods for MLVA information associated with this designation).
